# CD4 T cell therapy counteracts inflammaging and senescence by preserving gut barrier integrity

**DOI:** 10.1126/sciimmunol.adv0985

**Published:** 2025-08-01

**Authors:** Manuel M. Gómez de las Heras, Elisa Carrasco, Mario Pérez-Manrique, Naohiro Inohara, Sandra Delgado-Pulido, Álvaro Fernández-Almeida, María I. Gálvez-Castaño, Isaac Francos-Quijorna, Carolina Simó, Virginia García-Cañas, J. Ignacio Escrig-Larena, Juan Francisco Aranda, Gonzalo Soto-Heredero, Enrique Gabandé-Rodríguez, Eva María Blanco, Joyce Días-Almeida, Gabriel Núñez, María Mittelbrunn

**Affiliations:** 1 Tissue and Organ Homeostasis Program, Centro de Biologia Molecular Severo Ochoa (CBM), Consejo Superior de Investigaciones Científicas (CSIC) - Universidad Autónoma de Madrid (UAM); Madrid, Spain; 2 Department of Molecular Biology, Faculty of Sciences, Universidad Autónoma de Madrid (UAM); Madrid, Spain; 3 Department of Biology, Faculty of Sciences, Universidad Autónoma de Madrid (UAM); Madrid, Spain; 4 Instituto Universitario de Biología Molecular-IUBM, Universidad Autónoma de Madrid (UAM); Madrid, Spain; 5 Instituto Ramón y Cajal de Investigación Sanitaria (IRYCIS); Madrid, Spain; 6 Department of Pathology and Rogel Cancer Center, University of Michigan Medical School; Ann Arbor, MI, USA; 7 Molecular Nutrition and Metabolism, Institute of Food Science Research (CIAL), Consejo Superior de Investigaciones Científicas (CSIC) - Universidad Autónoma de Madrid (UAM); Madrid, Spain; 8 Department of Genetics, Physiology and Microbiology, Faculty of Biological Sciences, Complutense University of Madrid (UCM); Madrid, Spain; 9 Center for Infectious Disease Education and Research (CiDER); Osaka University, Suita, Osaka, Japan

## Abstract

Healthy aging relies on a symbiotic host–microbiota relationship. The age-associated decline of the immune system can pose a threat in this delicate equilibrium. In this work, we investigated how the functional deterioration of T cells can impact host–microbiota symbiosis and gut barrier integrity and the implications of this deterioration for inflammaging, senescence, and health decline. Using the *Tfam*^fl/fl^*Cd4*^Cre^ mouse model, we found that T cell failure compromised gut immunity leading to a decrease in T follicular and regulatory T (T_reg_) cells and an accumulation of highly proinflammatory and cytotoxic T cells. These alterations were associated with intestinal barrier disruption and gut dysbiosis. Microbiota depletion or adoptive transfer of total CD4 T cells or a T_reg_ cell–enriched pool prevented gut barrier dysfunction and mitigated premature inflammaging and senescence, ultimately enhancing healthspan in this mouse model. Thus, a competent CD4 T cell compartment is critical to ensure healthier aging by promoting host–microbiota mutualism and gut barrier integrity.

## Introduction

The intimate crosstalk between the immune system and the intestinal environment is critical to ensure mutualism and to prevent gut inflammation and disease (1). The age-related functional decline of immune cells poses a potential threat to this delicate equilibrium. During aging, CD4 T cells progressively accumulate in the intestine acquiring highly proinflammatory and cytotoxic profiles (2, 3). Moreover, gut-associated germinal centers (GCs) become defective due to imbalanced T follicular and B cell activity, which is accompanied by the expansion of IgA-producing plasma cells in the lamina propria and dysregulated IgA responses (2–6). Targeting of the gut microbiota by IgA is severely altered during aging, which can impact the composition of these microbial communities driving dysbiosis (5, 6). This dysbiosis compromises the integrity of the gut barrier, which consequently promotes an inflammatory state in the intestine and the systemic dissemination of bacterial products fueling the chronic inflammation that appears with aging, a process referred to as inflammaging (7, 8). Gut dysbiosis and intestinal barrier dysfunction are currently proposed to be hallmarks of aging (9–11), although their role in the promotion of age-associated pathologies remains poorly understood.

Emerging evidence reveals the contribution of immune cells, with T cells in the spotlight, as drivers of inflammaging and the subsequent accumulation of senescent cells in peripheral tissues, a process thought to play a role in the onset of age-related disorders (12–15). Although different mechanisms have been proposed (16), the process by which a defective T cell compartment contributes to inflammaging, senescence, and disease is still unclear. To address this question, we used the *Tfam*^fl/fl^*Cd4*^Cre^ mouse model, which carries a deficiency in the mitochondrial transcription factor A (TFAM) in both CD4 and CD8 T cells (17). TFAM is implicated in the maintenance and replication of mitochondrial DNA and its deletion accelerates the age-associated mitochondrial decline that appears in T cells. TFAM-deficient T cells foster premature inflammaging, tissue senescence, and aging-related disorders, altogether precipitating the death of these mice (12). In this work, we sought to investigate whether dysfunctional T cells accelerate inflammaging, senescence, and health decline through the loss of host–microbiota symbiosis and the disruption of the intestinal barrier. We find that depletion of the microbiota or restoration of regulatory T (T_reg_) cells prevents gut dysbiosis and intestinal barrier dysfunction, which mitigates systemic inflammaging and tissue senescence in *Tfam*^fl/fl^*Cd4*^Cre^ mice, ultimately improving health outcomes in this mouse model.

## Results

### Intestinal barrier integrity is severely compromised in *Tfam*^fl/fl^*Cd4*^Cre^ mice

**Fig. 1:**
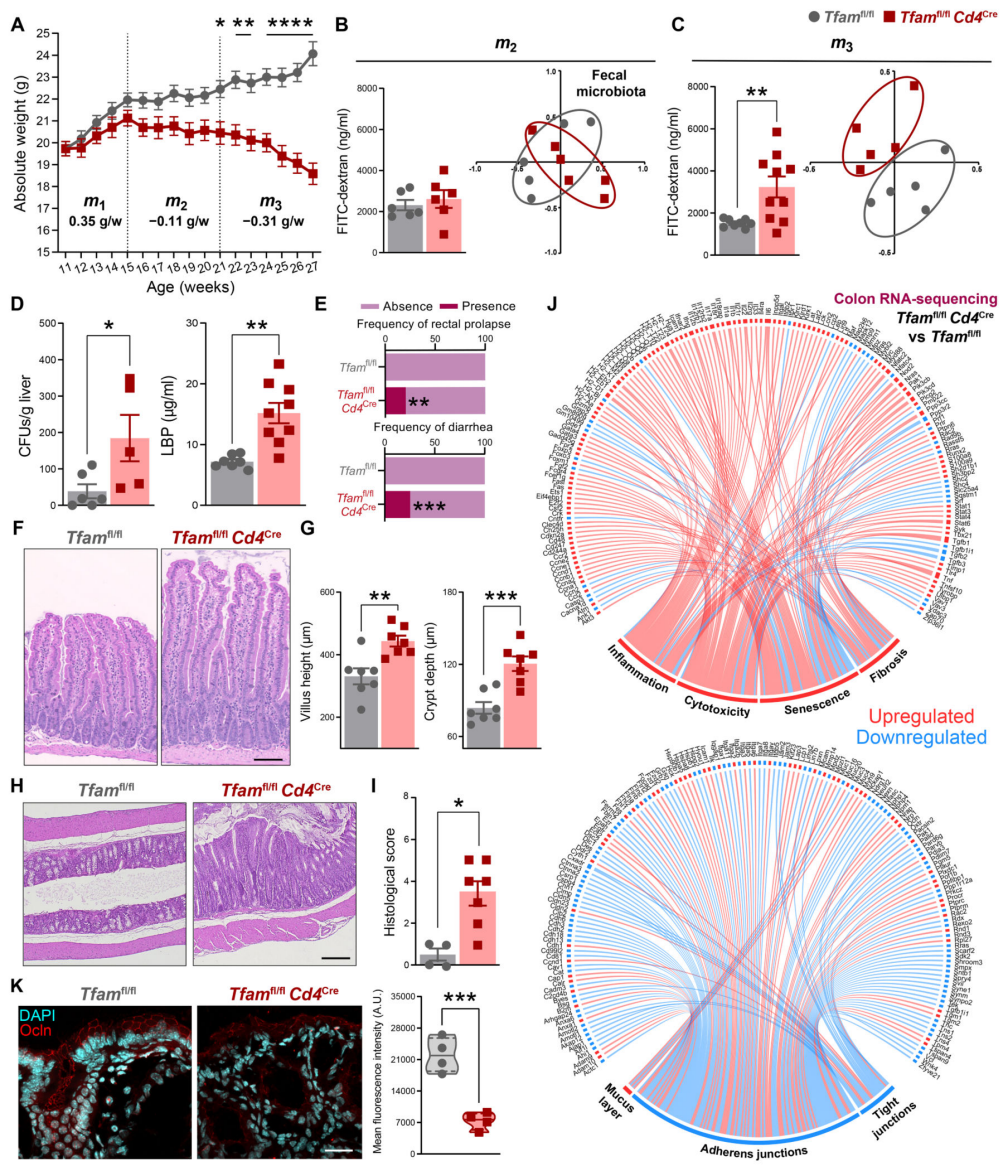
Intestinal barrier integrity is compromised in *Tfam*^fl/fl^*Cd4*^Cre^ micex. (**A**) Longitudinal assessment of body weight in *Tfam*^fl/fl^ and *Tfam*^fl/fl^*Cd4*^Cre^ mice, where *m* denotes the slope of the regression line (*n* = 3 to 8). (**B** and **C**) Concentration of FITC-dextran in the serum (*n* = 4 to 6) and nonmetric multidimensional scaling (NMDS) plots of β-diversity (θYC indexes) in fecal microbiota (*n* = 5 to 6) during (B) *m_2_* and (C) *m_3_* phases. (**D**) Quantification of colony-forming units (CFUs) per gram of liver (*n* = 2 to 3) and levels of LPS-binding protein (LBP) in the serum of 12-month-old mice (*n* = 3 to 5). (**E**) Percentage of mice displaying rectal prolapse or diarrhea (*n* = 3 to 8). (**F**) Representative hematoxylin and eosin (H&E)–stained sections of the small intestine (scale bar: 120 μm). (**G**) Quantification of villus height and crypt depth in the small intestine (*n* = 7). (**H**) Representative H&E-stained sections of the colon (scale bar: 100 μm). (**I**) Pathological scoring of colon histology (*n* = 4 to 7). (**J**) Chord diagrams of RNA-sequencing analysis representing up-regulated (red) or down-regulated (blue) genes in the colons of *Tfam*^fl/fl^*Cd4*^Cre^ versus *Tfam*^fl/fl^ mice. (**K**) Representative image (scale bar: 20 μm) and quantification of occludin (Ocln) immunofluorescence staining in the colon (*n* = 4). Data are pooled from (A and E) *N* = 7 to 8 or (C and D) *N* = 2 to 4 independent experiments. Data are shown as means ± SEM, where each dot is a biological sample. *P* values were determined by (A) mixed-effects analysis with Šidák’s multiple comparison test, (D, G, and K) unpaired Student’s *t* test, (E) Fisher’s exact test, or (I) two-tailed Mann–Whitney *U* test. (B and C) *P* values were determined by unpaired Student’s *t* test (bar graphs) or permutational multivariate analysis of variance (PERMANOVA) (NMDS plots). *P ≤ 0.05; **P ≤ 0.01; ***P ≤ 0.001; and ****P ≤ 0.0001.

Since intestinal barrier integrity and gut microbiota play a relevant role in health and disease (7, 9, 11), we investigated whether gut barrier disruption mediates the multimorbidity phenotype of *Tfam*^fl/fl^*Cd4*^Cre^ mice. Although these mice develop normally until approximately 4 to 8 months of age, they begin to lose weight at that moment correlating with the appearance of the multimorbidity syndrome (12). We observed two different stages of this syndrome: an initial mild and progressive loss of body weight (*m_2_* = −0.11 g per week) was followed by wasting disease characterized by an acute weight loss (*m_3_* = −0.31 g per week) that preceded the deaths of these mice (Fig. 1A). Only *Tfam*^fl/fl^*Cd4*^Cre^ mice in *m_3_* phase, but not earlier, showed enhanced gut permeability as assessed after oral administration of FITC-dextran as well as alterations in the composition of fecal microbiota compared to control littermates (Fig. 1, B and C). Leaky gut and gut dysbiosis were also accompanied by markers of bacterial translocation to the periphery, as determined by increased presence of culturable bacteria in the liver and heightened amounts of LPS-binding protein (LBP) in the serum of *Tfam*^fl/fl^*Cd4*^Cre^ mice compared to age-matched controls (Fig. 1D). Since approximately 20% of adult *Tfam*^fl/fl^*Cd4*^Cre^ mice displayed rectal prolapse and approximately 25% developed diarrhea at the steady state (Fig. 1E), we next explored intestinal pathology. Histological analysis of the small intestine in *Tfam*^fl/fl^*Cd4*^Cre^ mice revealed an altered tissue architecture with an increase in villus height and crypt depth accompanied by mild edema and inflammatory cell infiltrates in the lamina propria (Fig. 1, F and G). Although no differences were observed in the length of the small intestine or the colon (fig. S1A), the colons of *Tfam*^fl/fl^*Cd4*^Cre^ mice were macroscopically thicker compared to what observed in control littermates (fig. S1B). Moreover, histological examination of the colon showed edematous swelling of the mucosa, with enlarged crypts containing inflammatory cell infiltrates and a thickened muscular layer, which was associated with a worsened histological score in this mouse model (Fig. 1, H and I).

RNA sequencing analysis of colonic tissue from *Tfam*^fl/fl^*Cd4*^Cre^ mice and control littermates revealed an enrichment in pathways related to inflammation, immune cell–mediated cytotoxicity, senescence, and fibrosis (Fig. 1J). Moreover, *Tfam*^fl/fl^*Cd4*^Cre^ mice exhibited an overall down-regulation in the expression of genes involved in the architecture and regulation of cell-to-cell junctional complexes (Fig. 1J). qPCR analysis further corroborated a strong proinflammatory and fibrotic signature in both the small intestine and colon of *Tfam*^fl/fl^*Cd4*^Cre^ mice (fig. S1C), together with reduction in the expression of genes involved in tight junctions of the small intestine (i.e., *Cldn1*) and the colon (i.e., *Tjp1* and *Ocln*), as well as in genes encoding antimicrobial peptides (fig. S1, D and E). The diminished expression of *Tjp1* and *Ocln*, encoding zonula occludens (ZO)-1 and occludin, respectively, translated into reduced immunofluorescence staining and disorganized structure of these anchoring proteins in the colonic epithelium compared to control littermates, evidencing disruption of the intestinal barrier (Fig. 1K and fig. S1F). Thus, the multimorbidity phenotype of *Tfam*^fl/fl^*Cd4*^Cre^ mice is associated with the loss of gut barrier integrity and subsequent endotoxemia.

### *Tfam*^fl/fl^*Cd4*^Cre^ mice display bacterial dysbiosis in the intestine

**Fig. 2:**
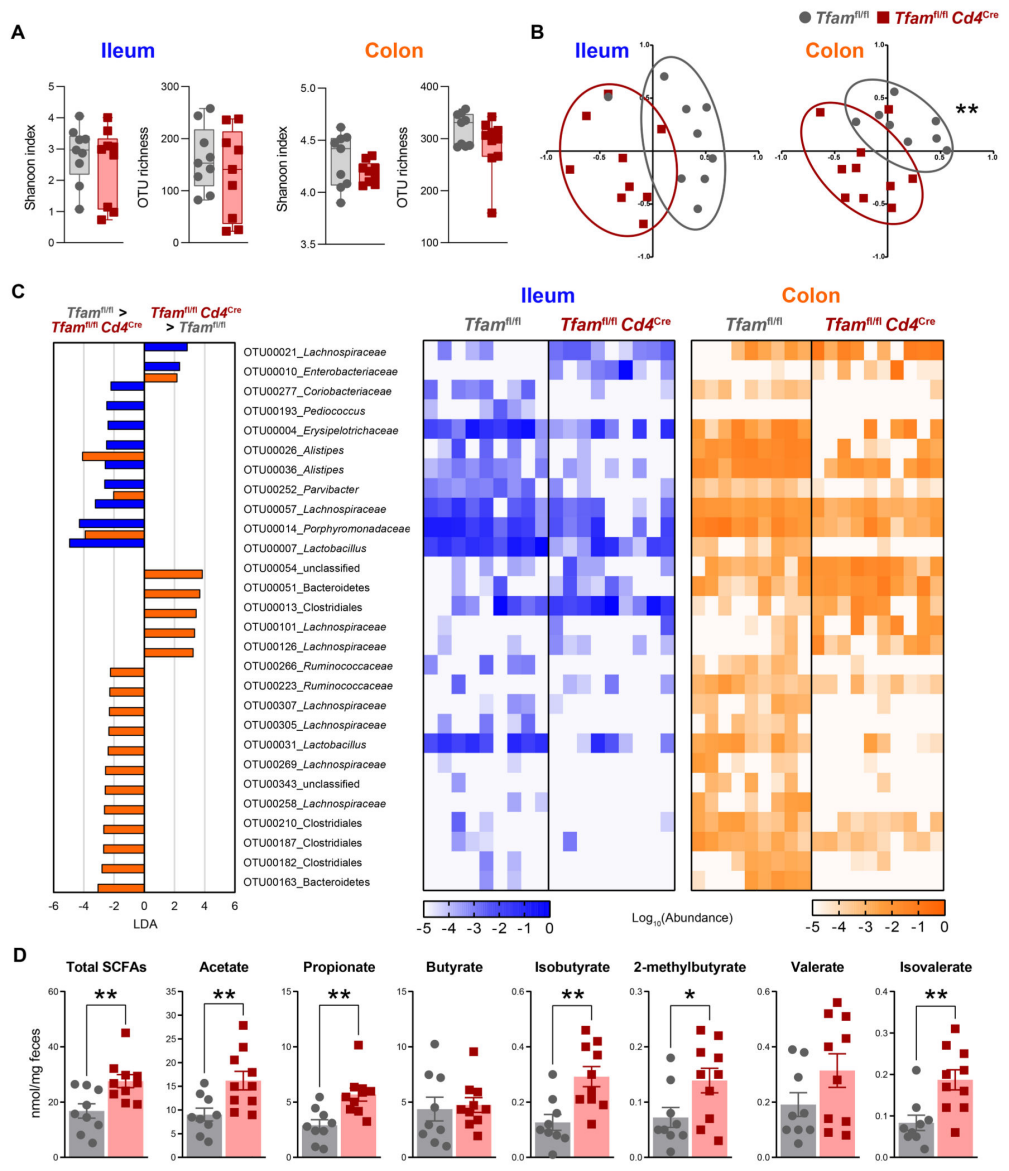
*Tfam*^fl/fl^*Cd4*^Cre^ mice display an inflammation-biased gut microbiota with exacerbated production of SCFAs. (**A**) Shannon index, and operational taxonomic unit (OTU) richness parameters of α-diversity in the ileum and colon-resident microbiota from 12-month-old *Tfam*^fl/fl^ and *Tfam*^fl/fl^*Cd4*^Cre^ mice (*n* = 4 to 5). (**B**) Non-metric multidimensional scaling (NMDS) plots showing β-diversity values (θYC indexes) of microbiota in the ileum and colon (*n* = 4 to 5). (**C**) Left: differentially abundant OTUs depicted with linear discriminant analysis (LDA) values of linear discriminant effect size (LEfSe, *P* < 0.05; false discovery rate, *q* < 0.05; fold change > 5; maximal abundance > 0.001) comparing ileal and colonic microbiota in *Tfam*^fl/fl^*Cd4*^Cre^ versus *Tfam*^fl/fl^ mice. Right: heatmap depicting abundance values. (**D**) Quantification of short-chain fatty acids (SCFAs) in the feces (*n* = 4 to 5). (A to D) Data are pooled from *N* = 2 independent experiments. Data are shown as means ± SEM, where each dot is a biological sample. *P* values were determined by (A) two-tailed Mann–Whitney *U* test, (B) permutational multivariate analysis of variance (PERMANOVA), or (D) unpaired Student’s *t* test. *P ≤ 0.05 and **P ≤ 0.01.

To further characterize the bacterial communities in both the small and large intestine of *Tfam*^fl/fl^*Cd4*^Cre^ mice during the *m_3_* phase, we performed *16S* rRNA gene sequencing from the luminal content of the terminal ileum and the colon. Analysis of α-diversity indexes such as Shannon index or operational taxonomic unit (OTU) richness, which disclose the diversity of species within each sample, showed no differences in the ileum or colon-resident microbiota (Fig. 2A). By contrast, analysis of β-diversity stating the similarity in terms of biodiversity between samples, revealed a distinct configuration of microbiota in the ileum and colon of *Tfam*^fl/fl^*Cd4*^Cre^ mice compared to control littermates (Fig. 2B). We next analyzed the microbial compositional changes in both intestinal compartments. There was a large reduction in health-promoting symbionts in the guts of *Tfam*^fl/fl^*Cd4*^Cre^ mice compared to control littermates including certain members of the *Lactobacillus* genus (ileum and colon) and some members of the *Ruminococcaceae* family (colon) (Fig. 2C). These changes were accompanied by a substantial expansion of microorganisms associated with intestinal inflammation, such as members of the *Enterobacteriaceae* family (Fig. 2C).

We further explored the *16S* rRNA metagenomic data by performing a predictive functional analysis of the gut microbiome. These results suggested enrichment in pathways associated with biosynthesis of nucleosides/nucleotides, amino acids, and enzymatic cofactors in the colonic microbiota of *Tfam*^fl/fl^*Cd4*^Cre^ mice compared to controls (fig. S2A). Moreover, we found an increase in pathways related to the catabolism of amino acids and polysaccharides (i.e., urea cycle and carboxylate degradation, respectively) and to cellular bioenergetics such as the tricarboxylic acid (TCA) cycle compared to the microbiota of control littermates (fig. S2A).

Protein and polysaccharide catabolism by gut microbiota results in the balanced production of essential metabolites such as short-chain fatty acids (SCFAs), which beneficially impact host physiology (18). To determine whether the observed compositional changes in gut microbiota caused a disbalance in the abundance of these metabolites, we quantified the levels of SCFAs in fecal samples of this mouse model by using liquid chromatography–mass spectrometry (LC–MS). The concentration of most of these metabolites, but notably not butyrate, increased in *Tfam*^fl/fl^*Cd4*^Cre^ mice compared to control littermates (Fig. 2D). To gain some insight into which bacteria could be modulating these SCFA profiles, we performed Spearman correlation analysis between the composition of the ileum and colon microbiota and the concentration of SCFA species in the same samples. We found three OTUs belonging to Bacteroidetes in the colon, *Lachnospiraceae* in the ileum, and *Enterobacteriaceae* in both the ileum and colon that were abnormally expanded in *Tfam*^fl/fl^*Cd4*^Cre^ mice and positively correlated with the enhanced production of SCFAs (fig. S2B). Thus, *Tfam*^fl/fl^*Cd4*^Cre^ mice manifest dysbiosis in the intestine featured by expansion of inflammation-related taxa and disproportioned levels of SCFAs.

### Microbiota depletion prevents inflammaging and tissue senescence extending health and lifespan in *Tfam*^fl/fl^*Cd4*^Cre^ mice

**Fig. 3:**
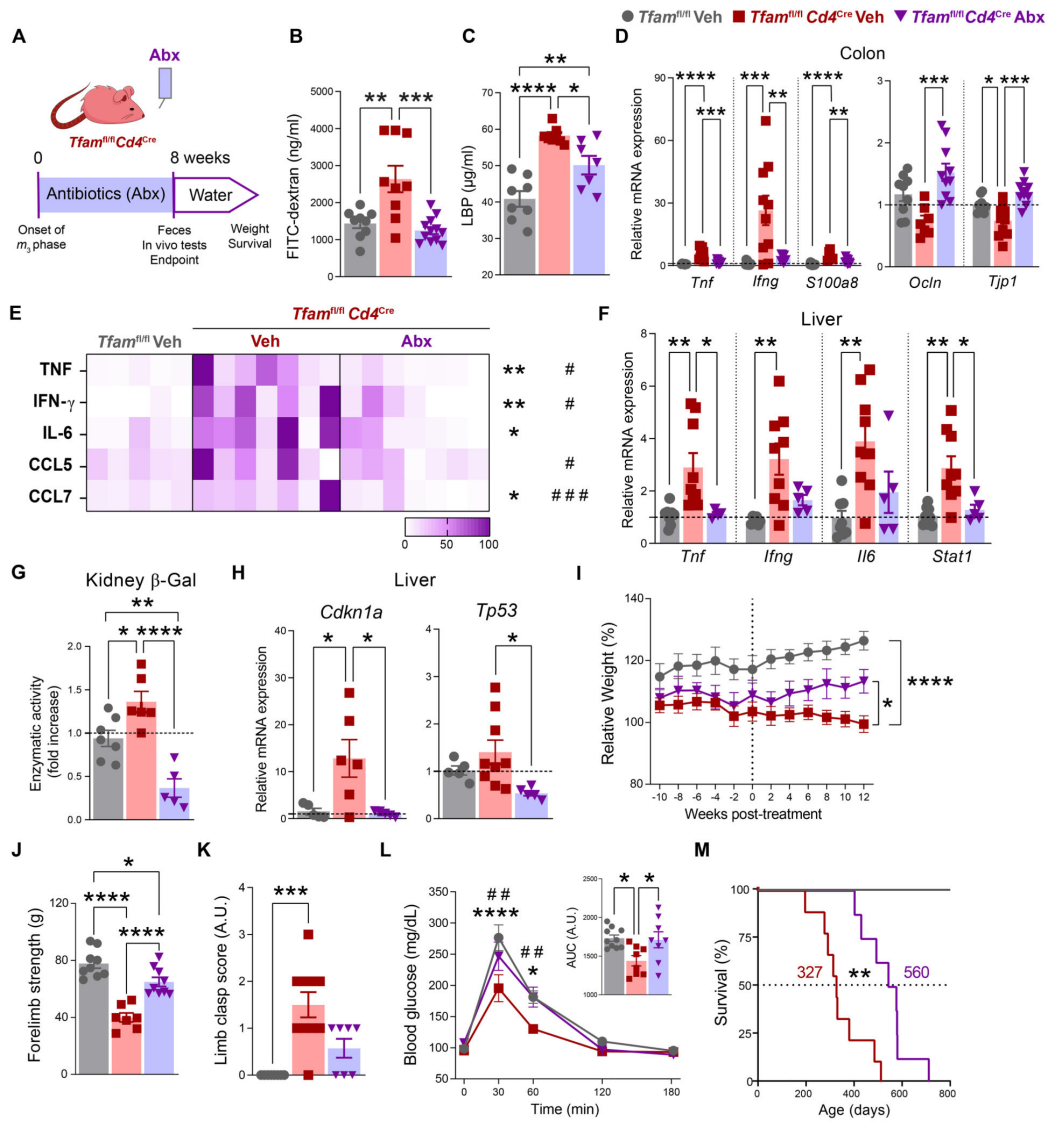
Microbiota depletion reinforces gut barrier integrity preventing inflammaging, senescence, and multimorbidity in *Tfam*^fl/fl^*Cd4*^Cre^ mice. (**A**) Experimental design of antibiotic-induced microbiota depletion. (**B**) Concentration of FITC-dextran in the serum of 12-month-old *Tfam*^fl/fl^ and *Tfam*^fl/fl^*Cd4*^Cre^ mice treated with antibiotics (Abx) or vehicle (Veh) (*n* = 4 to 5). (**C**) Levels of LPS-binding protein (LBP) in the serum (*n* = 3 to 4). (**D**) Relative mRNA levels of genes associated with inflammation (*Tnf, Infg*, and *s100a8*) or with tight junctions (*Ocln* and *Tjp1*) in the colon (*n* = 3 to 5). (**E**) Heatmap depicting normalized concentration of inflammatory mediators in the serum (*n* = 5 to 7). (**F**) Relative mRNA levels of genes associated with inflammation (*Tnf, Infg, Il6*, and *Stat1*) in the liver (*n* = 3 to 6). (**G**) Quantification of β-galactosidase (β-Gal) activity in kidney lysates (*n* = 2 to 5). (**H**) Relative mRNA levels of the senescence-associated genes *Cdkn1a* and *Tp53* in the liver (*n* = 3 to 5). (**I**) Body weight relative to the beginning of the treatment (*n* = 3 to 6). (**J**) Grip test (*n* = 4 to 5). (**K**) Clasping score (*n* = 4). (**L**) Glucose tolerance test and its area under the curve (AUC) (*n* = 3 to 4). (**M**) Kaplan–Meier survival curves (*n* = 4 to 5). (B to M) Data are pooled from *N* = 2 independent experiments. Data are shown as means ± SEM, where each dot is a biological sample. *P* values were determined by (B to H, and J) one-way analysis of variance (ANOVA) with Tukey’s multiple comparisons test, (I) two-way ANOVA with Šidák’s multiple comparison test, (K) Kruskal–Wallis *H* test with Dunn’s multiple comparison test, or (M) log-rank (Mantel–Cox) test. (L) *P* values were determined by one-way (curve) or two-way ANOVA (AUC) with Tukey’s multiple comparisons test. *Tfam*^fl/fl^ versus *Tfam*^fl/fl^*Cd4*^Cre^ (*)**;** *Tfam*^fl/fl^*Cd4*^Cre^ versus *Tfam*^fl/fl^*Cd4*^Cre^ Abx (^#^). *^,#^*P* ≤ 0.05; **^,##^*P* ≤ 0.01; ***P ≤ 0.001; and ****P ≤ 0.0001.

To decipher the contribution of gut dysbiosis in the multimorbidity phenotype of *Tfam*^fl/fl^*Cd4*^Cre^ mice, we depleted microbiota in these mice using a cocktail of broad-spectrum antibiotics (Abx) for 8 weeks upon the onset of the *m_3_* phase (Fig. 3A). This protocol successfully reduced the presence of bacteria, as well as the concentration of SCFAs in feces of Abx-treated mice (fig. S3, A to C). Administration of Abx did not induce any substantial changes either in the body weight (fig. S3D) or survival of control mice.

Depletion of dysbiotic microbial communities in *Tfam*^fl/fl^*Cd4*^Cre^ mice strengthened the integrity of the gut barrier as shown by reduced penetration of FITC-dextran after oral administration, concomitantly with diminished bacterial translocation to the periphery (Fig. 3, B and C). Macroscopic evaluation of the colon suggested that Abx administration to *Tfam*^fl/fl^*Cd4*^Cre^ mice restrained thickening and fibrosis at this level (fig. S3E). To further assess the integrity of the intestinal barrier, we evaluated markers of inflammation and cell-to-cell junctional complexes in the intestine of *Tfam*^fl/fl^*Cd4*^Cre^ mice after Abx administration. Microbiota depletion suppressed the inflammatory transcriptomic signature found in the ileum and the colon of *Tfam*^fl/fl^*Cd4*^Cre^ mice and up-regulated the levels of transcripts encoding proteins involved in the physical integrity of the intestine such as claudin-1 in the ileum and occludin and ZO-1 in the colon compared to mice receiving vehicle (Fig. 3D and fig. S3F).

Furthermore, *Tfam*^fl/fl^*Cd4*^Cre^ mice treated with Abx displayed diminished levels of inflammatory mediators in the serum, for instance, C-C motif chemokine ligand 5 (CCL5) and CCL7, interferon (IFN)-γ, interleukin (IL)-6, and tumor necrosis factor (TNF), as well as reduced levels of proinflammatory markers in the liver (Fig. 3, E and F) suggesting that the development of inflammaging was being prevented. This was accompanied by decreased signs of kidney and liver senescence, as evidenced by reduced senescence-associated β-galactosidase activity (Fig. 3G) and down-regulated expression of the senescence-associated genes encoding P21^Waf/Cip1^ and P53, respectively (Fig. 3H). Importantly, microbiota depletion prevented the acute loss of body weight at the onset of the multimorbidity phenotype of *Tfam*^fl/fl^*Cd4*^Cre^ mice (Fig. 3I). Muscle strength and locomotor coordination assessed by grip test and clasping score, respectively, were also improved after Abx administration (Fig. 3, J and K). Glucose tolerance tests showed that glucose homeostasis in *Tfam*^fl/fl^*Cd4*^Cre^ mice was restored to the levels of control mice after depletion of microbiota (Fig. 3L). Finally, there was a 42% extension in the median survival and approximately 30% in the maximal survival of Abx-treated *Tfam*^fl/fl^*Cd4*^Cre^ mice compared to mice receiving vehicle (Fig. 3M). Thus, depletion of the dysbiotic microbiota in *Tfam*^fl/fl^*Cd4*^Cre^ mice prevents gut barrier dysfunction and diminishes the levels of inflammaging and tissue senescence leading to an extended health and lifespan in these mice.

To evaluate the pathogenicity of *Tfam*^fl/fl^*Cd4*^Cre^ mouse gut microbiota, we performed fecal microbiota transplantation (FMT) assays from either donor *Tfam*^fl/fl^*Cd4*^Cre^ at the onset of the *m_3_* phase or age-matched *Tfam*^fl/fl^ mice into young antibiotic-treated *Tfam*^fl/fl^ mice (referred to as *Tfam*^fl/fl^FMT_KO_ or *Tfam*^fl/fl^FMT_Ctrl_ mice, respectively) (fig. S4A). Analysis of the fecal microbiota showed successful implantation of donor microbiota in recipient mice (fig. S4B). Nevertheless, we did not find any differences in terms of body weight, muscle strength, or gut barrier integrity, with absent or minimal impact in the levels of proinflammatory cytokines in the serum of *Tfam*^fl/fl^FMT_KO_ mice when compared to *Tfam*^fl/fl^FMT_Ctrl_ mice (fig. S4, C to H). Thus, the gut microbiota from *Tfam*^fl/fl^*Cd4*^Cre^ mice may not be sufficient to cause inflammaging and tissue damage in control mice harboring a competent immune system.

### Gut mucosal immunity is impaired in *Tfam*^fl/fl^*Cd4*^Cre^ mice

Since CD4 T cells are essential for host–microbiota symbiosis and gut barrier function, we explored CD4 T cell subsets in Peyer’s patches (PPs) and the colonic lamina propria (cLP) of adult (12-month-old) *Tfam*^fl/fl^*Cd4*^Cre^ mice and age-matched controls by using a panel of antibodies for spectral flow cytometry that decodes the heterogeneity of T cells.

**Fig. 4:**
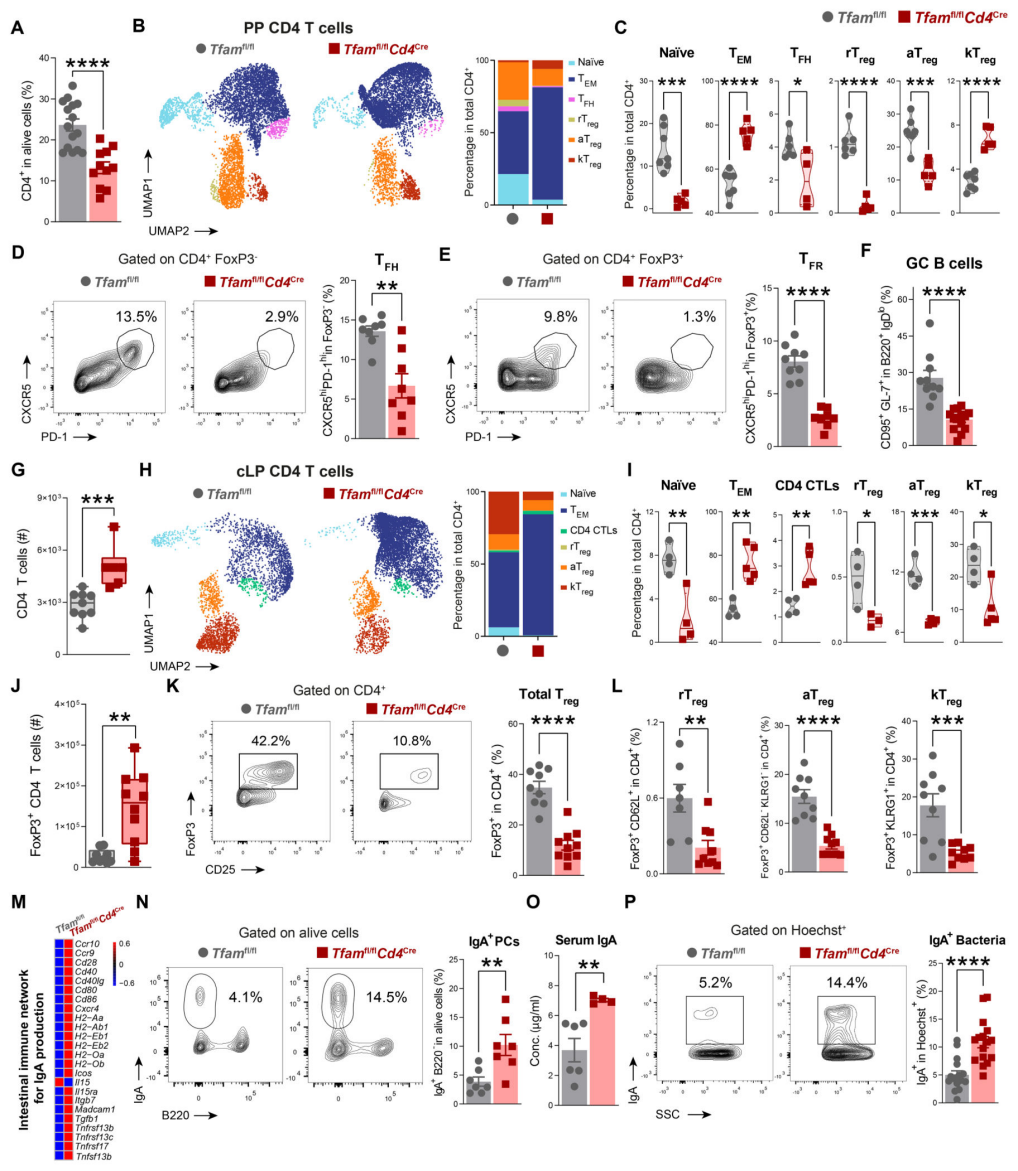
Gut mucosal immunity is impaired in *Tfam*^fl/fl^*Cd4*^Cre^ mice. (**A**) Percentage of CD4 T cells in Peyer’s patches (PPs) of 12-month-old *Tfam*^fl/fl^ and *Tfam*^fl/fl^*Cd4*^Cre^ mice (*n* = 3 to 7). (**B**) Left: representative UMAP showing PP CD4 T cell clusters. Right: bar plots showing the percentage of clusters. (**C**) Percentage of PP clusters (*n* = 4 to 7). (**D** and **E**) Representative contour plots and quantification of (D) T follicular helper (T_FH_) and (E) T follicular regulatory (T_FR_) cells in PPs (*n* = 3 to 5). (**F**) Quantification of germinal center (GC) B cells in PPs (*n* = 3 to 5). (**G**) Absolute number of CD4 T cells in the colonic lamina propria (cLP) (*n* = 4 to 5). (**H**) Left: representative UMAP showing cLP CD4 T cell clusters. Right: bar plots showing the percentage of clusters. (**I**) Percentage of cLP clusters (*n* = 3 to 5). (**J**) Absolute number of cLP regulatory T (T_reg_) cells (*n* = 4 to 6). (**K**) Representative contour plots and quantification of cLP T_reg_ cells (*n* = 4 to 6). (**L**) Quantification of resting (r), activated (a), and KLRG1^+^ (k)T_reg_ cells in the cLP (*n* = 3 to 6). (**M**) Heatmap depicting expression of genes related to IgA biosynthesis in the colon RNA-sequencing analysis. (**N**) Representative contour plot and quantification of cLP IgA^+^ plasma cells (PCs) (*n* = 7). (**O**) Concentration of serum IgA (*n* = 4 to 7). (**P**) Representative contour plot and quantification of IgA-coated fecal bacteria (*n* = 4 to 7). Data are (B, C, H, and I) representative of *N* = 2 to 3 independent experiments or (A, D to F, J to L, O, and P) pooled from *N* = 2 to 3 independent experiments. Data are shown as means ± SEM, where each dot is a biological sample. *P* values were determined by unpaired Student’s *t* test. *P ≤ 0.05; **P ≤ 0.01; ***P ≤ 0.001; and ****P ≤ 0.0001.

Analysis of PPs showed that the percentage of CD4 T cells was substantially reduced in *Tfam*^fl/fl^*Cd4*^Cre^ mice (Fig. 4A and fig. S5A). Unbiased clusterization of PP CD4 T cells resulted in the identification of six subsets: naïve (FoxP3^−^CD62L^hi^CD44^lo^), T effector/memory-like (T_EM_) (FoxP3^−^CD62L^lo^CD44^hi^), T follicular helper (T_FH_) (FoxP3^−^CXCR5^hi^PD-1^hi^), and three different clusters of FoxP3^+^CD25^+^ T_reg_ cells, namely resting (rT_reg_) (CD62L^hi^CD44^lo^), activated (aT_reg_) (CD62L^lo^CD44^hi^), and a subset of effector/terminally differentiated T_reg_ cells (19, 20) expressing the killer cell lectin-like receptor G1 (KLRG1) (kT_reg_) cells, previously associated with aging (21) (Fig. 4B and fig. S6, A and B). The T_EM_ and kT_reg_ cell clusters were increased at the expense of a strong reduction in the naïve, T_FH_, rT_reg_, and aT_reg_ cell clusters in the PP CD4 T cell compartment of *Tfam*^fl/fl^*Cd4*^Cre^ mice (Fig. 4C). The percentage and absolute numbers of both T_FH_ and FoxP3^+^CXCR5^hi^PD-1^hi^ T follicular regulatory (T_FR_) cells were substantially reduced in *Tfam*^fl/fl^*Cd4*^Cre^ mice compared to control littermates (Fig. 4, D and E, and figs. S5A and S6C). The drop in T follicular cells was associated with a substantial reduction in the percentage and absolute numbers of mature CD95^+^GL-7^+^ B cells in the PPs of *Tfam*^fl/fl^*Cd4*^Cre^ mice (Fig. 4F and figs. S5A and S6D), suggesting a defect in gut-associated germinal center (GC) reactions. Although most of the alterations in CD4 T cell subsets and GC B cells in PPs of 12-month-old *Tfam*^fl/fl^*Cd4*^Cre^ mice resembled what was observed in 24-month-old wild-type mice, the frequencies of total CD4 T cells, aT_reg_ cells, and T_FR_ cells were all increased in aged wild-type mice (fig. S7, A to F).

Analysis of the cLP revealed an increase in the number of infiltrating CD4 T cells in *Tfam*^fl/fl^*Cd4*^Cre^ mice compared to controls (Fig. 4G and fig. S5B). By applying the same approach used in the PPs analysis, we found that the percentage of naïve CD4 T cells was reduced in the cLP of these mice, whereas CD4 T cells were skewed towards a highly activated and cytotoxic phenotype (CD4 cytotoxic T lymphocyte (CTL)) that produced large amounts of CCL5 at the steady state (Fig. 4, H and I, and fig. S6, E and F). To further explore the activity of TFAM-deficient T cells, we assessed the capacity of cLP CD4 T cells to produce proinflammatory cytokines by flow cytometry. Ex vivo stimulation experiments indicated that cLP CD4 T cells of *Tfam*^fl/fl^*Cd4*^Cre^ mice secreted more TNF and IFN-γ than those from control mice (fig. S6G). Although the absolute number of T_reg_ cells was increased in the cLP of *Tfam*^fl/fl^*Cd4*^Cre^ mice (Fig. 4J), the proportion of total T_reg_ cells and their various subsets were greatly reduced in the cLP CD4 T cell compartment of these mice (Fig. 4, I, K, and L, and fig. S5B), consistent with previous reports delineating the T_reg_ cell–specific requirements of mitochondrial metabolism (22, 23). Although 12-month-old *Tfam*^fl/fl^*Cd4*^Cre^ mice mirrored most of the changes observed in the cLP of 24-month-old wild-type mice, the frequencies of total T_reg_, aT_reg_, and kT_reg_ cells substantially increased in 24-month-old wild-type mice instead of decreasing as in *Tfam*^fl/fl^*Cd4*^Cre^ mice (fig. S7, G to K).

Since TFAM deletion also affects CD8 T cells in *Tfam*^fl/fl^*Cd4*^Cre^ mice due to a CD4^+^CD8^+^ double-positive state during T cell maturation (17), we next analyzed CD8 T cells in PPs and the cLP of these mice. Similar to what was observed in PP CD4 T cells, 12-month-old *Tfam*^fl/fl^*Cd4*^Cre^ mice replicated the drop in naïve CD8 T cells and the increase in T_EM_ and KLRG1-expressing CD8 T cells that was observed in 24-month-old wild-type mice (fig. S8, A to D). Moreover, there was increased infiltration of CD8 T cells into the cLP of *Tfam*^fl/fl^*Cd4*^Cre^ mice. These cells exhibited highly activated, cytotoxic, and regulatory profiles, as is observed in natural aging (fig. S8, E to H).

Because RNA sequencing analysis of the colon from *Tfam*^fl/fl^*Cd4*^Cre^ mice revealed that one of the most up-regulated pathways was the intestinal immune network for IgA production (Fig. 4M), we investigated whether the dysfunctional T cell compartment in the intestine of *Tfam*^fl/fl^*Cd4*^Cre^ mice was accompanied by dysregulated IgA responses. Similar to naturally aged mice (2), *Tfam*^fl/fl^*Cd4*^Cre^ mice displayed an increased percentage of IgA^+^ plasma cells (PCs) in the cLP (Fig. 4N and fig. S5B). This accumulation of IgA-producing PCs was associated with increased concentrations of IgA antibodies in the serum and enhanced IgA responses against the gut microbiota in the steady state compared to control littermates (Fig. 4, O and P). Thus, mitochondrial dysfunction appears to shift T cells towards highly inflammatory profiles with loss of follicular and regulatory subsets, which then abrogates GC reactions and dysregulates IgA responses in the intestines of *Tfam*^fl/fl^*Cd4*^Cre^ mice.

### T_reg_ cell therapy prevents inflammaging and senescence by restoring gut barrier integrity in Tfam^fl/fl^*Cd4*^Cre^ mice

**Fig. 5:**
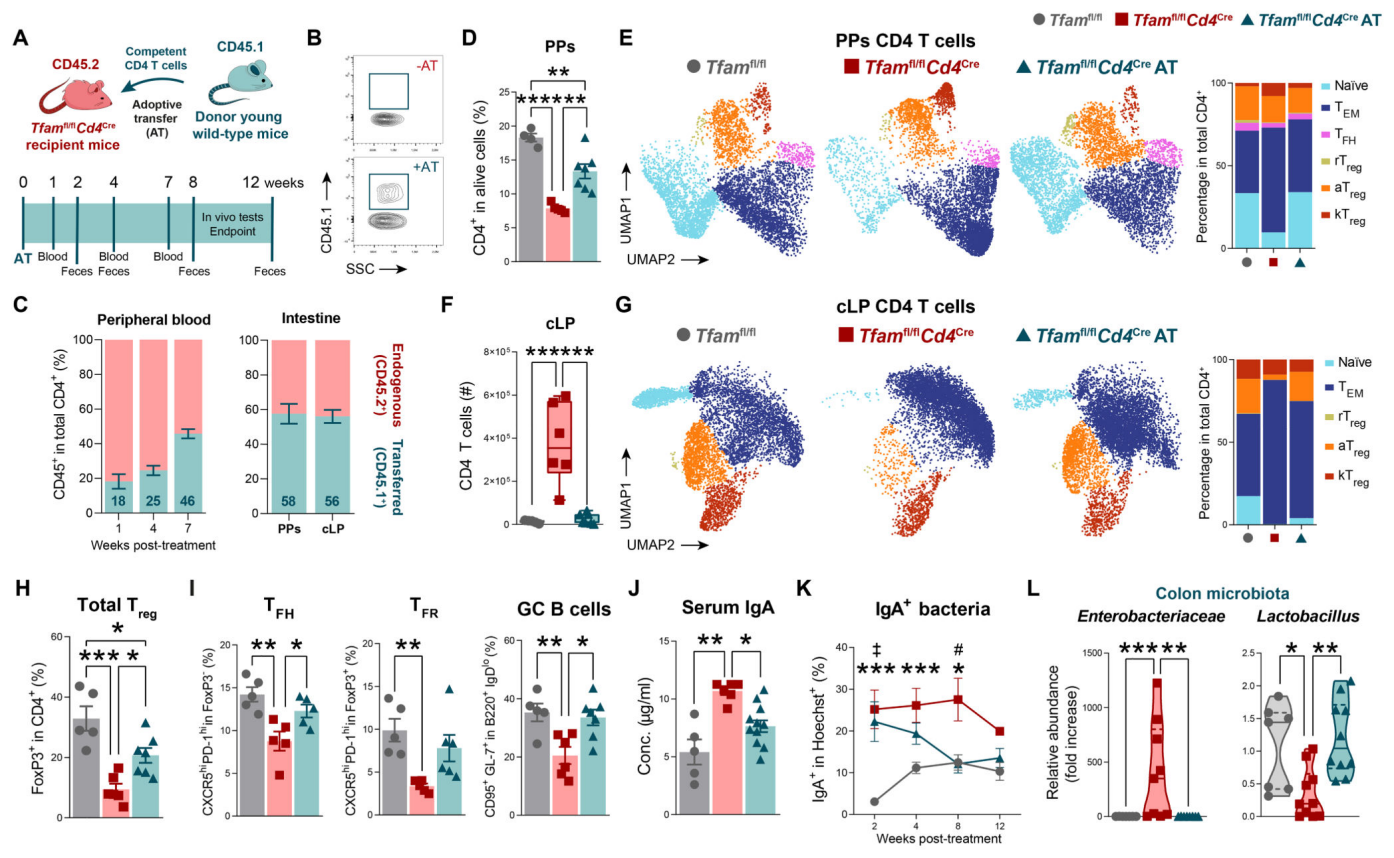
Adoptive transfer of CD4 T cells restores gut mucosal immunity in *Tfam*^fl/fl^*Cd4*^Cre^ mice. (**A**) Experimental design of CD4 T cell adoptive transfer (AT). (**B**) Donor cells in the peripheral blood of transferred and non-transferred 10-month-old *Tfam*^fl/fl^*Cd4*^Cre^ mice. (**C**) Mean chimeric ratio in the peripheral blood, Peyer’s patches (PPs), and the colonic lamina propria (cLP). (**D**) Percentage of PP CD4 T cells in *Tfam*^fl/fl^ and *Tfam*^fl/fl^*Cd4*^Cre^ mice after the transfer of CD4 T cells (*n* = 5 to 7). (**E**) Left: representative UMAP showing PP CD4 T cells clusters. Right: bar plots showing the percentage of clusters. (**F**) Absolute number of cLP CD4 T cells (*n* = 5 to 7). (**G**) Left: representative UMAP showing cLP CD4 T cell clusters. Right: bar plots showing the percentage of clusters. (**H**) Percentage of cLP regulatory T (T_reg_) cells (*n* = 5 to 7). (**I**) Quantification of T follicular helper (T_FH_) and regulatory (T_FR_) cells and germinal center (GC) B cells in PPs (*n* = 5 to 7). (**J**) Concentration of serum IgA (*n* = 4 to 7). (**K**) Longitudinal quantification of IgA-coated fecal bacteria (*n* = 3 to 7). (**L**) qPCR quantification of colon-resident microbiota (*n* = 3 to 7). Data are (B to I) representative of *N* = 2 or (J to L) pooled from *N* = 2 independent experiments. Data are shown as means ± SEM, where each dot is a biological sample. *P* values were determined by (D, F, H, and I) one-way analysis of variance (ANOVA) with Tukey’s multiple comparisons test, (J and L) Kruskal–Wallis *H* test with Dunn’s multiple comparison test, and (K) mixed-effects analysis with Tukey’s multiple comparisons test. *Tfam*^fl/fl^ versus *Tfam*^fl/fl^*Cd4*^Cre^ (*)**;** *Tfam*^fl/fl^*Cd4*^Cre^ versus *Tfam*^fl/fl^*Cd4*^Cre^ AT (^#^); *Tfam*^fl/fl^ versus *Tfam*^fl/fl^*Cd4*^Cre^ AT (^‡^). *^,#,‡^*P* ≤ 0.05; **P ≤ 0.01; ***P ≤ 0.001; and ****P ≤ 0.0001.

Given the considerable remodeling of the T cell compartment in the intestines of *Tfam*^fl/fl^*Cd4*^Cre^ mice, we next tested whether the replenishment of key CD4 T cell subsets would safeguard gut homeostasis and, by extension, prevent the multimorbidity syndrome in this mouse model. We therefore adoptively transferred competent CD4 T cells from young CD45.1^+^ mice into CD45.2^+^ *Tfam*^fl/fl^*Cd4*^Cre^ recipient mice, prior to *m_3_* phase (Fig. 5, A and B). By week 7 after the adoptive transfer, CD4 T cells from donors reached 46% of total circulating CD4 T cells in recipient mice (Fig. 5C). Two months after the adoptive transfer, donor CD45.1^+^ CD4 T cells comprised nearly 60% of total CD4 T cells in PPs and the cLP of these mice (Fig. 5C).

Analysis of PPs showed that the percentage of total CD4 T cells was normalized in recipient *Tfam*^fl/fl^*Cd4*^Cre^ mice almost to the levels of control mice (Fig. 5D). Unbiased clusterization of spectral flow cytometry data unveiled that the frequency of different subsets in the whole CD4 T cell compartment was restored because of this adoptive therapy. These changes comprised the recovery of the naïve, T follicular, rT_reg_, and aT_reg_ cell pools, as well as a marked decline in the percentage of T_EM_ and kT_reg_ cells (Fig. 5E and fig. S9, A to E). Furthermore, the excessive infiltration of CD4 T cells was completely reverted in *Tfam*^fl/fl^*Cd4*^Cre^ mice transferred with competent CD4 T cells compared to their controls (Fig. 5F), further restoring the pool of naïve and T_EM_ CD4 T cells (Fig. 5G and fig. S9, F to J). CD4 T cell therapy significantly replenished the pool of T_reg_ cells in the colon of *Tfam*^fl/fl^*Cd4*^Cre^ mice by almost duplicating the proportion of cLP T_reg_ cells (Fig. 5H and fig. S9, H to J). The recovery of CD4 T cell homeostasis in the intestine of transferred *Tfam*^fl/fl^*Cd4*^Cre^ mice was associated with a boost in gut-associated GC reactions (Fig. 5I) and rebalanced IgA responses, evidenced by reduced concentration of circulating IgA antibodies and diminished opsonization of intestinal bacteria over time after the adoptive transfer (Fig. 5, J and K). CD4 T cell adoptive therapy consistently restored eubiosis in the colon of recipient mice featured by reduction in the relative abundance of inflammation-related *Enterobacteriaceae* and increase in *Lactobacillus* sp. (Fig. 5L).

**Fig. 6:**
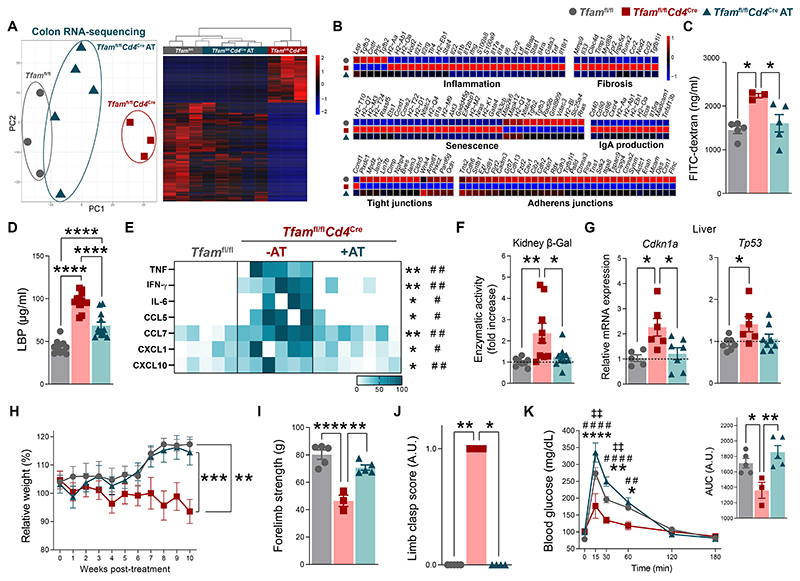
CD4 T cell therapy prevents inflammaging, senescence, and multimorbidity by restoring gut barrier integrity in *Tfam*^fl/fl^*Cd4*^Cre^ mice. (**A**) Left: principal component analysis (PCA) of colon RNA-sequencing data. Right: hierarchical clustering of differentially expressed genes. (**B**) Heatmaps depicting expression of genes in the colon RNA-sequencing analysis. (**C**) Concentration of FITC-dextran in the serum (*n* = 3 to 5). (**D**) Quantification of LPS-binding protein (LBP) in the serum (*n* = 5 to 7). (**E**) Heatmap depicting normalized concentration of inflammatory mediators in the serum (*n* = 5 to 7). (**F**) Quantification of β-galactosidase (β-Gal) activity in kidney lysates (*n* = 3 to 7). (**G**) Bar plots showing relative mRNA levels of the senescence-associated genes *Cdkn1a* and *Tp53* in the liver (*n* = 3 to 5). (**H**) Body weight relative to the beginning of the treatment (*n* = 3 to 6). (**I**) Grip test (*n* = 3 to 5). (**J**) Clasping score (*n* = 3 to 4). (**K**) Glucose tolerance test and area under the curve (AUC) quantification (*n* = 3 to 5). Data are (C, E, and I to K) representative of *N* = 2 or (D, F to H) pooled from *N* = 2 independent experiments. Data are shown as means ± SEM, where each dot is a biological sample. *P* values were determined by (C to G, and I) one-way analysis of variance (ANOVA) with Tukey’s multiple comparisons test, (H) mixed-effects analysis with Tukey’s multiple comparisons test, (J) Kruskal–Wallis *H* test with Dunn’s multiple comparison test. (K) *P* values were determined by one-way (AUC) or two-way ANOVA (curve) with Tukey’s multiple comparisons test. *Tfam*^fl/fl^ versus *Tfam*^fl/fl^*Cd4*^Cre^ (*)**;** *Tfam*^fl/fl^*Cd4*^Cre^ versus *Tfam*^fl/fl^*Cd4*^Cre^ AT (^#^); *Tfam*^fl/fl^ versus *Tfam*^fl/fl^*Cd4*^Cre^ AT (^‡^). *^,#^*P* ≤ 0.05; **^,##,‡‡^*P* ≤ 0.01; ***P ≤ 0.001; and ****^,####^*P* ≤ 0.0001.

Macroscopic examination of the colon suggested that CD4 T cell therapy to *Tfam*^fl/fl^*Cd4*^Cre^ mice restrained thickening at this level (fig. S9K). To explore wider effects of restoring immune homeostasis in the intestine of these mice, we performed RNA sequencing of colon samples. Principal component analysis and hierarchical clustering of the transcriptomic data showed that samples from *Tfam*^fl/fl^*Cd4*^Cre^ mice transferred with competent CD4 T cells clustered closer to control than to non-transferred *Tfam*^fl/fl^*Cd4*^Cre^ mice, suggesting that CD4 T cell adoptive therapy favored the reversion of the transcriptional changes observed in the colon of this mouse model (Fig. 6A). In particular, we found that the expression levels of genes involved in inflammation, immune cell–mediated cytotoxicity, IgA biosynthesis, senescence, and fibrosis were down-regulated compared to non-transferred *Tfam*^fl/fl^*Cd4*^Cre^ mice, whereas the expression levels of genes related to cell-to-cell junction organization were increased as a result of the adoptive transfer (Fig. 6B and fig. S9L). Consistent with these results, ZO-1 and occludin immunofluorescence staining showed a partial restoration of the expression pattern of these proteins in the colonic epithelium of adoptively transferred *Tfam*^fl/fl^*Cd4*^Cre^ mice (fig. S9M). Furthermore, gut hyperpermeability and bacterial translocation were prevented in *Tfam*^fl/fl^*Cd4*^Cre^ mice after the adoptive transfer (Fig. 6, C and D), indicating a reinforcement of gut barrier integrity.

CD4 T cell therapy also diminished the levels of the inflammaging mediators IFN-γ, IL-6 and TNF, as well as those of several chemokines such as CCL5, CCL7, C-X-C motif chemokine ligand 1 (CXCL1), and CXCL10 in the serum of recipient *Tfam*^fl/fl^*Cd4*^Cre^ mice (Fig. 6E). This was accompanied by a reduction in signs of tissue senescence, namely restored levels of senescence-associated β-galactosidase activity in the kidney and reduced expression of the senescence-associated genes encoding P21^Waf/Cip1^ and P53 in the liver of recipient mice (Fig. 6, F and G). Adoptively transferred *Tfam*^fl/fl^*Cd4*^Cre^ mice showed an improvement in multiple signs of multimorbidity including the marked loss of body weight, muscle atrophy, locomotor disability or glucose dysregulation (Fig. 6, H to K), suggesting that the healthspan of these mice had been enhanced.

**Fig. 7:**
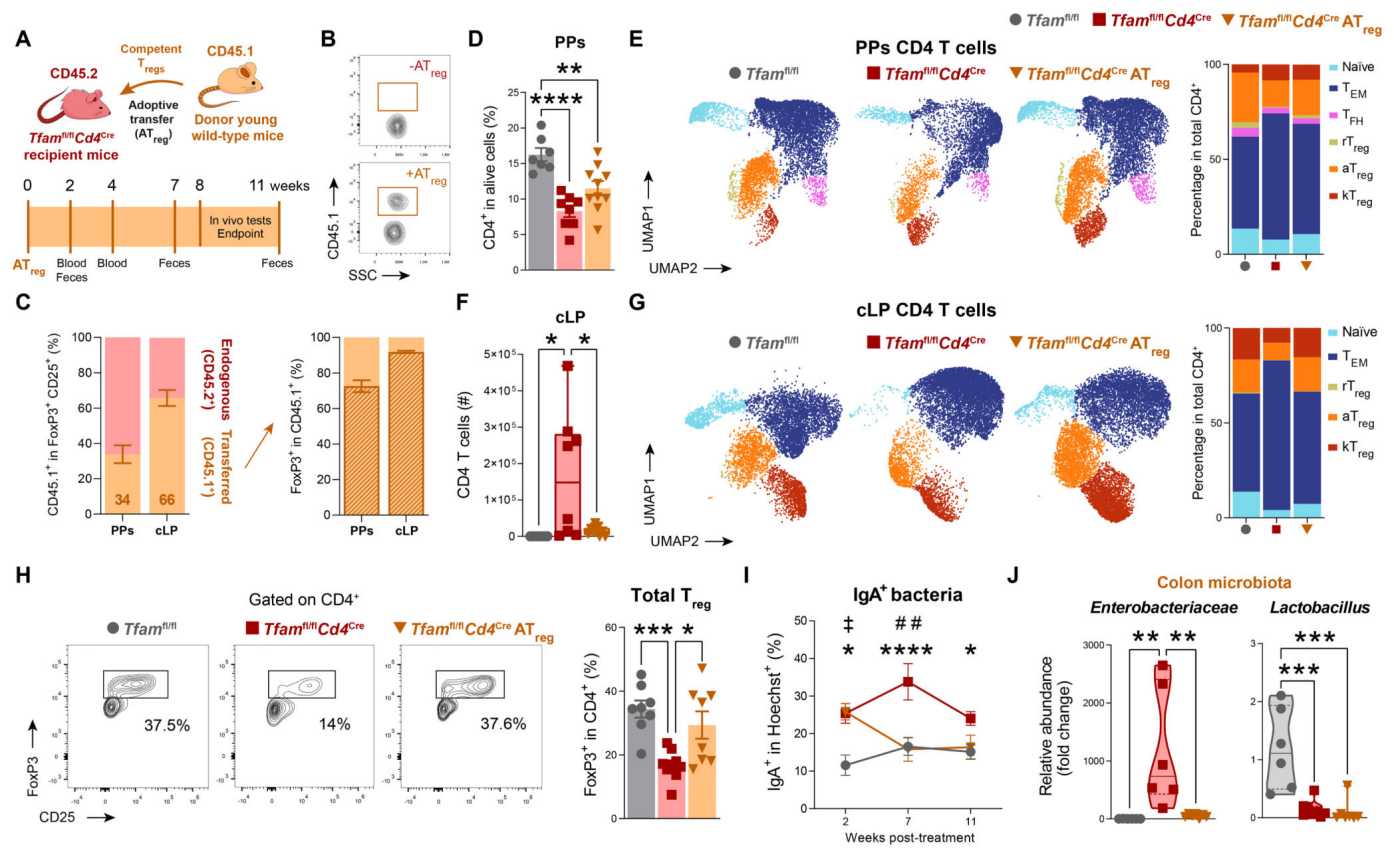
Adoptive therapy of a T_reg_ cell–enriched pool rebalances gut immune homeostasis in *Tfam*^fl/fl^*Cd4*^Cre^ mice. (**A**) Experimental design of regulatory T (T_reg_) cell adoptive transfer (AT_reg_). (**B**) Donor cells in the peripheral blood of transferred and non-transferred 10-month-old *Tfam*^fl/fl^*Cd4*^Cre^ mice. (**C**) Mean chimeric ratio and percentage of FoxP3^+^ donor cells in Peyer’s patches (PPs) and the colonic lamina propria (cLP). (**D**) Percentage of PP CD4 T cells in *Tfam*^fl/fl^ and *Tfam*^fl/fl^*Cd4*^Cre^ mice after the transfer of a T_reg_ cell–enriched pool (*n* = 4 to 6). (**E**) Left: representative UMAP showing PP CD4 T cells. On Right: bar plots showing the percentage of clusters. (**F**) Absolute number of cLP CD4 T cells (*n* = 3 to 6). (**G**) Left: representative UMAP showing cLP CD4 T cell clusters. Right: bar plots showing the percentage of clusters. (**H**) Representative contour plots and quantification of cLP T_reg_ cells (*n* = 3 to 6). (**I**) Longitudinal quantification of IgA-coated fecal bacteria (*n* = 3 to 6). (**J**) qPCR quantification of colon-resident microbiota (*n* = 3 to 5). Data are (B, C, E, and G) representative of *N* = 2 or (D, F, and H to J) pooled from *N* = 2 independent experiments. Data are shown as means ± SEM, where each dot is a biological sample. *P* values were determined by (D, F, H, and J) one-way analysis of variance (ANOVA) with Tukey’s multiple comparisons test or (I) mixed-effects analysis with Tukey’s multiple comparisons test. *Tfam*^fl/fl^ versus *Tfam*^fl/fl^*Cd4*^Cre^ (*)**;** *Tfam*^fl/fl^*Cd4*^Cre^ versus *Tfam*^fl/fl^*Cd4*^Cre^ AT_reg_ (^#^); *Tfam*^fl/fl^ versus *Tfam*^fl/fl^*Cd4*^Cre^ AT_reg_ (^‡^). *^,‡^*P* ≤ 0.05; **^,##^*P* ≤ 0.01; ***P ≤ 0.001; and ****P ≤ 0.0001.

**Fig. 8:**
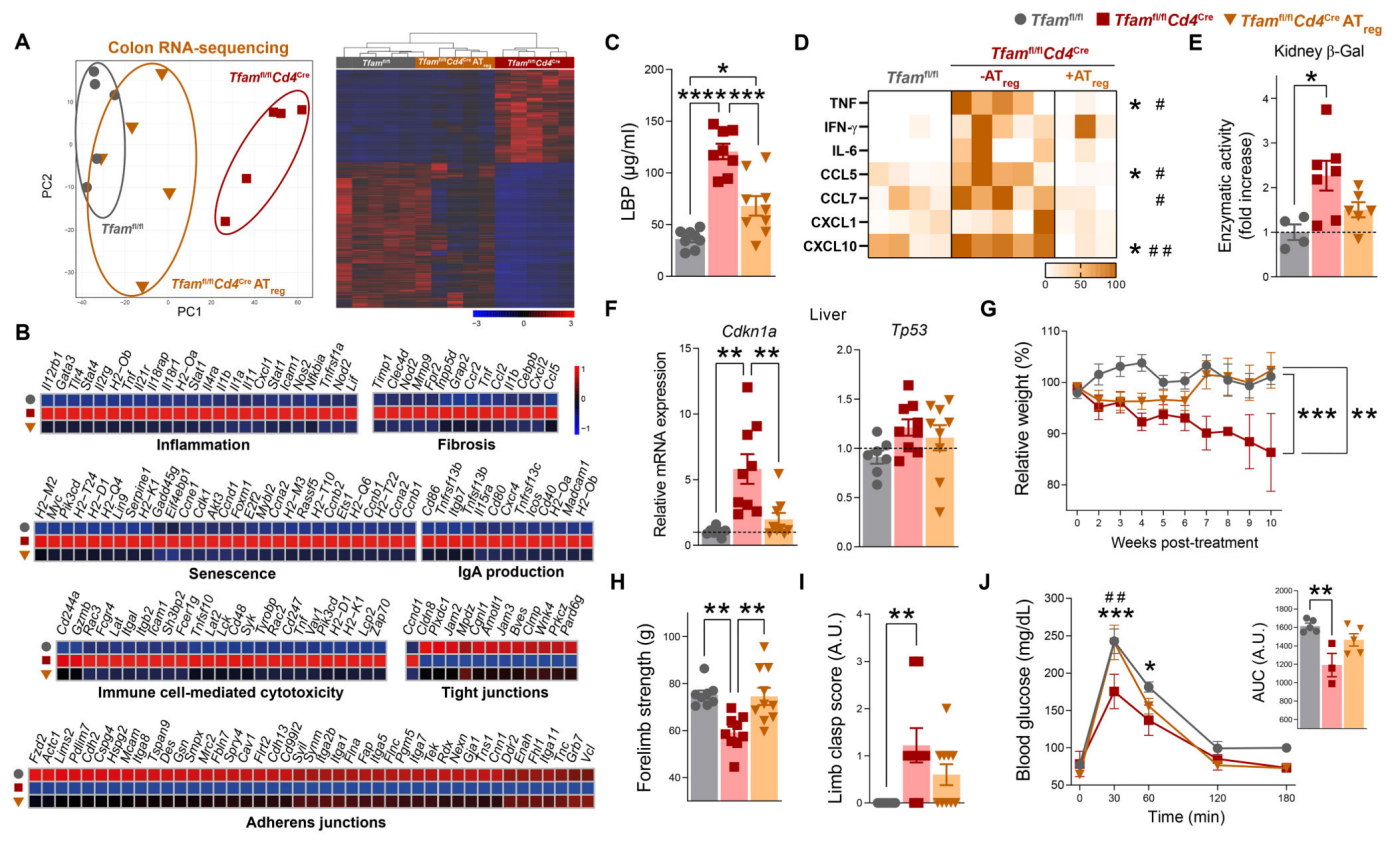
Transfer of a T_reg_ cell–enriched pool restores gut barrier integrity preventing inflammaging, senescence, and multimorbidity in *Tfam*^fl/fl^*Cd4*^Cre^ mice. (**A**) Left: principal component analysis (PCA) of colon RNA-sequencing data. Right: hierarchical clustering of differentially expressed genes. (**B**) Heatmap depicting expression of genes in the colon RNA-sequencing analysis. (**C**) Quantification of LPS-binding protein in the serum (*n* = 3 to 6). (**D**) Heatmap depicting normalized concentration of inflammatory mediators in the serum (*n* = 3 to 5). (**E**) Quantification of β-galactosidase (β-Gal) activity in kidney lysates (*n* = 2 to 4). (**F**) Bar plots showing relative mRNA levels of the senescence-associated genes *Cdkn1a* and *Tp53* in the liver (*n* = 3 to 6). (**G**) Body weight relative to the beginning of the treatment (*n* = 3 to 6). (**H**) Grip test (*n* = 3 to 6). (**I**) Clasping score (*n* = 3 to 6). (**J**) Glucose tolerance test and area under the curve (AUC) quantification (*n* = 3 to 5). Data are (C, and E to I) pooled from *N* = 2 or (A, B, D, and J) representative of *N* = 2 independent experiments. Data are shown as means ± SEM, where each dot is a biological sample. *P* values were determined by (C to F, and H) one-way analysis of variance (ANOVA) with Tukey’s multiple comparisons test, (G) mixed-effects analysis with Tukey’s multiple comparisons test, or (I) Kruskal-Wallis *H* test with Dunn’s multiple comparison test. (J) *P* values were determined by (AUC) one-way ANOVA or (curve) two-way ANOVA with Tukey’s multiple comparisons test. *Tfam*^fl/fl^ versus *Tfam*^fl/fl^*Cd4*^Cre^ (*); and *Tfam*^fl/fl^*Cd4*^Cre^ versus *Tfam*^fl/fl^*Cd4*^Cre^ AT_reg_ (^#^). *P ≤ 0.05; **^,##^*P* ≤ 0.01; ***P ≤ 0.001; and ****P ≤ 0.0001.

To further understand which subset of CD4 T cells was crucial for the recovery of *Tfam*^fl/fl^*Cd4*^Cre^ mice as a result of the adoptive therapy, we performed adoptive transfer of a T_reg_ cell–enriched CD4 T cell pool from young CD45.1^+^ mice into CD45.2^+^ *Tfam*^fl/fl^*Cd4*^Cre^ recipient mice, successfully replenishing the intestinal compartment (Fig. 7, A to C, and fig. S10A). This strategy also reestablished immune balance in the intestine of recipient mice, notably increasing the frequency of rT_reg_, aT_reg_, and T_FR_ cells in PPs, as well as total T_reg_, aT_reg_, and kT_reg_ cells in the cLP of transferred mice (Fig. 7, D to H, and fig. S10, B to L). All of these changes were associated with improved GC B cell reactions and rebalanced IgA responses against intestinal bacteria (Fig. 7I and fig. S10F). Adoptive transfer of a T_reg_ cell–enriched pool also prevented the expansion of *Enterobacteriaceae* in the colon (Fig. 7J), and restrained gut barrier disruption, evidenced by RNA-sequencing analysis of the colon and diminished bacterial translocation to the periphery (Fig. 8, A to C, and fig. S10M). Moreover, adoptive transfer of a T_reg_ cell–enriched pool prevented inflammaging and tissue senescence (Fig. 8, D to F), as well as the acute body weight loss, sarcopenia, and dysregulation of glucose handling in transferred *Tfam*^fl/fl^*Cd4*^Cre^ mice (Fig. 8, G to J) evidencing an improved health status in this mouse model.

Finally, we investigated whether the CD8 T cell compartment was also affected after these adoptive transfer strategies. Analysis of PPs showed that the increased frequency of KLRG1-expressing CD8 T cells was prevented in transferred *Tfam*^fl/fl^*Cd4*^Cre^ mice after both adoptive transfer strategies. Adoptive transfer of either CD4 T cells or a T_reg_ cell–enriched pool limited both excessive CD8 T cell infiltration as well as the prominent expansion of KLRG1^+^ and regulatory CD8 T cell subsets in the cLP of transferred *Tfam*^fl/fl^*Cd4*^Cre^ mice (fig. S11, E to H). This suggests that CD8 T cells may also participate in the maintenance of gut barrier integrity. Thus, the transfer of CD4 T cells—T_reg_ cells in particular—restores the intestinal immune balance of *Tfam*^fl/fl^*Cd4*^Cre^ mice, which in turn prevents gut dysbiosis and strengthens gut barrier integrity, ultimately enhancing healthspan in this mouse model.

## Discussion

The Nobel laureate Elie Metchnikoff proposed a century ago that the decline in health that occurs during aging is driven by increased systemic inflammation resulting from the rupture of the intestinal barrier and subsequent dissemination of pathogenic bacteria (24). This theory received limited attention from the scientific community until pioneering studies in the field showed that gut barrier dysfunction predicts imminent health decline and death in flies (25). Since then, evidence supporting the idea that the deterioration of this biological barrier is a conserved hallmark in the process of unhealthy aging has accumulated in species such as nematodes (26), fish (27), mice (7, 8, 28), monkeys (29, 30), and humans (31). However, the underlying causes are still under debate.

T cells undergo a dramatic remodeling during aging mainly comprising the accumulation of terminally differentiated T cell subsets at the expense of losing naïve/resting T cells (32). Similar to other cell types in the organism, the mitochondrial function of T cells deteriorates during aging (12, 21, 33). We mimicked this age-associated mitochondrial decline in T cells by depleting TFAM specifically in CD4 and CD8 T cells. T cell mitochondrial failure in *Tfam*^fl/fl^*Cd4*^Cre^ mice accelerates some of the hallmarks of T cell aging such as lysosomal dysfunction and lack of T cell plasticity (17) or severe immunodeficiency (12), but also fosters inflammaging, tissue senescence, and multiple aging-related disorders (12). We previously discussed that different mechanisms could cooperate to explain the contribution of dysfunctional T cells to inflammaging and senescence (16). Dysfunctional T cells, for example, can directly damage the tissues via the secretion of inflammatory mediators (12, 34). In addition, impaired immunosurveillance can indirectly increase the burden of senescent cells in the tissues (35, 36). These cells also lack regenerative potential, which is essential to maintain tissue homeostasis (37, 38). In this work, we provide evidence for an additional mechanism by which dysfunctional T cells contribute to inflammaging and senescence through the loss of their ability to control host–microbiota symbiosis and gut barrier integrity. Our data indicate that mitochondrial failure in T cells causes intestinal barrier disruption and gut dysbiosis, correlating with the development of multimorbidity syndrome prior to death in *Tfam*^fl/fl^*Cd4*^Cre^ mice. Although TFAM is deleted in both CD4 and CD8 T cells in the early stages of T cell development in *Tfam*^fl/fl^*Cd4*^Cre^ mice (17), transferring competent CD4 T cells—T_reg_ cells in particular—is sufficient to restore mucosal immunity in the intestine of these mice, which is associated with reinforcement of the gut barrier, prevention of gut dysbiosis, and delay of multiple signs of multimorbidity.

T_reg_ cells, which mainly depend on mitochondrial metabolism (22, 23), are particularly important to ensure homeostasis in the intestine serving as constrainers of inflammatory insults and promoters of tissue repair after damage (39). The T_reg_ cell compartment is augmented in lymphoid and nonlymphoid organs during aging (32) but, instead of exerting anti-inflammatory and pro-resolving roles, they become dysfunctional showing proinflammatory and damaging features (21, 40, 41). In contrast to the increased frequency of effector T_reg_ cells, including aT_reg_ cells and kT_reg_ cells, observed in the colons of naturally aged wild-type mice, we detected a substantial reduction in these T_reg_ cell subsets in the colons of *Tfam*^fl/fl^*Cd4*^Cre^ mice. This decrease was associated with enhanced inflammation and deterioration of the intestinal barrier. These results suggest that the loss of T_reg_ cell subpopulations may exacerbate a proinflammatory environment to an extent that is not observed in naturally aged wild-type mice, thereby defining a distinctive feature between natural aging and the accelerated aging shown by *Tfam*^fl/fl^*Cd4*^Cre^ mice. Our T_reg_ cell–enriched cell transfer experiments suggest that recovery of metabolically competent T_reg_ cells could palliate the premature aging phenotype of this mouse model triggered by TFAM deficiency in CD4 and CD8 T cell compartments. Furthermore, T_reg_ cells are essential for GC dynamics and the appropriate IgA control of gut microbiota to avoid dysbiosis and intestinal inflammation (42, 43). IgA is the predominant antibody in the intestinal mucosa and is generated either by a T cell–dependent mechanism—in which T follicular cells dictate the differentiation of mature GC B cells into PCs that produce high-affinity and high-specificity antibodies—or in a T cell–independent manner, whereby PCs produce low-affinity polyreactive antibodies (44). Our results indicate that adoptive transfer of a T_reg_ cell–enriched pool of CD4 T cells boosts GC reactions and normalizes the enhanced opsonization of intestinal bacteria by IgA antibodies in *Tfam*^fl/fl^*Cd4*^Cre^ mice. Research has shown that elevated levels of IgA coating uniquely identify colitogenic bacteria during gut inflammation (45). Transfer of a T_reg_ cell–enriched pool is consistently associated with reduced presence of proinflammatory bacteria in the gut microbiota of these mice. Thus, loss of T_reg_ cell subsets in the intestine of *Tfam*^fl/fl^*Cd4*^Cre^ mice could lead to dysregulated IgA responses that facilitate gut dysbiosis, altogether precipitating gut barrier disruption and fueling inflammaging in this mouse model. Accordingly, microbiota depletion prevented intestinal barrier dysfunction, which correlated with diminished inflammaging, tissue senescence, and health decline in *Tfam*^fl/fl^*Cd4*^Cre^ mice.

One limitation of this study is that approximately 5% of the transfer donor CD4 T cells were FoxP3^−^ cells in the T_reg_ cell–enriched cell transfer experiments. Donor-derived cells were subsequently detected within the T_EM_ and T_FH_ cell pools of recipient mice. This could be either due to the proliferation of contaminating donor FoxP3^−^ CD4 T cells—including donor T_FH_ cells—and/or the generation of productive T_FH_ cells from donor T_reg_ cells in the PPs of recipient mice as has been previously observed (46), which may account for the rescue in GC B cell activity we observed following adoptive transfer.

*Tfam*^fl/fl^*Foxp3*^Cre^ mice have a lifespan of 60 days due to lethal autoimmune disease (22), whereas *Tfam*^fl/fl^*Cd4*^Cre^ mice can live longer than 1.5 years (12). One plausible explanation may be that conventional T cells remain healthy in *Tfam*^fl/fl^*Foxp3*^Cre^ mice and the lack of functional T_reg_ cells is responsible for these dramatic consequences. In the case of *Tfam*^fl/fl^*Cd4*^Cre^ mice, both conventional and regulatory T cells are defective. However, since TFAM-deficient conventional T cells are unable to proliferate to a similar extent than wild-type cells (17), the reduction in T_reg_ cells leads to less drastic outcomes, although still contributing to gut barrier dysfunction. These results support the notion that there is a delicate equilibrium between conventional and T_reg_ cells in the context of tissue homeostasis and the murine healthspan.

T cell–based immunotherapies have recently emerged as an innovative approach to address age-related diseases. Chimeric antigen receptor (CAR) T cells designed to target senescent cells improved metabolic dysfunction and physical performance in mice during aging (47) and senescence overload (48). Adoptive transfer of T_reg_ cells from young mice limited retinal neurodegeneration and partially restored spinal cord remyelination in naturally aged mice (37, 49). Moreover, coadministration of T_reg_ cells upon transplantation of dopamine-producing neurons alleviated neuroinflammation in mouse models of Parkinson’s disease (50). Our findings indicate that T cell therapy mitigates systemic inflammaging and prevents senescence in several tissues (e.g., the colon, liver, and kidney) boosting resilience to multiple signs of multimorbidity in *Tfam*^fl/fl^*Cd4*^Cre^ mice. In conclusion, this work provides insights into the potential applications of T cell–based therapies to delay age-associated pathologies through the strengthening of intestinal barrier integrity.

## Materials and Methods

### Animal procedures

All animal experimentation procedures were authorized by the Animal Experimentation Ethics Committees of Centro de Biología Molecular Severo Ochoa (CBM) and Centro Superior de Investigaciones Científicas (ProEx 52.1/23) making every effort to minimize mouse discomfort. Wild-type (C57BL/6J HccRsd) and Ly5.1 (CD45.1) mice were either purchased from Envigo or generated and aged in the CBM Animal Facility. Wild-type, *Tfam*^fl/fl^ and *Tfam*^fl/fl^*Cd4*^Cre^ mice were bred, aged, and maintained in the CBM Animal Facility under specific pathogen-free conditions. Two to five mice were housed per cage separated by genotype and sex, and fed ad libitum, receiving cardboard materials as part of the environmental enrichment. Mice were considered young (≤ 3 months of age), adult (4 to 12 months of age) or aged (≥ 13 months of age) following The Jackson Laboratory’s guidelines. Most studies were performed using female mice to facilitate microbiota normalization by mice and/or cage swapping experiments were initiated.

For intestinal permeability experiments, mice fasted for 2 hours were orally gavaged with a 1:3-1:4 (v:v) dilution of 250 mg/ml of 4-kDa fluorescein isothiocyanate (FITC)-dextran probe (Sigma) at a dose of 0.6 g per kilogram of body weight. After 2 hours, 100 to 120 μl of blood was collected in BD Microtainer^®^ tubes from the facial vein of mice and centrifuged at 6000*g* for 10 min at 4°C to obtain the serum fraction. FITC-dextran measurements were performed in duplicates by fluorimetric quantification of mouse serum mixed with an equal volume of phosphate buffered saline (PBS) [1:9 (v:v)]. Dilutions of non-treated mouse serum in FITC-dextran diluted with PBS were used as a standard curve to calculate blood FITC-dextran concentrations. One hundred microliters of standards or diluted sera was measured in a FLUOstar OPTIMA^®^ (BMG Labtech) 96-plate reader at an excitation wavelength of 492 nm and an emission wavelength of 525 nm.

For antibiotics-induced microbiota depletion, mice were randomly assigned either to vehicle or antibiotics (Abx) treatment groups. Mice in the latter group were administered a cocktail of neomycin (1 mg/ml) (Nzytech), ampicillin (1 mg/ml) (Nzytech), metronidazole (1 mg/ml) (Sigma), and vancomycin (0.5 mg/ml) (Sigma) in autoclaved drinking water supplemented with sucrose (2 mg/ml) to improve palatability for 8 weeks. This solution was renewed once a week. Vehicle mice were treated with sucrose water without antibiotics for the same time period. Microbiota depletion was further corroborated by flow cytometry and *16S* rRNA gene qPCR quantification using bacterial DNA extracted from feces as further detailed.

For muscle strength measurements, mice were held by their tails and forelimb grip strength was measured as tension force using a digital force transducer (Grip Strength Meter, Bioseb), as previously described (12). Five to seven measurements per trial were performed for each mouse, with a few seconds resting period between measurements.

For hindlimb clasping score measurements, mice were suspended by their tails for 10 s and video-recorded, as previously described (51). In brief, mice received a score depending on the following criteria: both hindlimbs splayed outward away from the abdomen, with splayed toes (0 points); one hindlimb retracted towards the abdomen more than half of the time (1 points); both hindlimbs retracted towards the abdomen more than half of the time (2 points); or both hindlimbs fully retracted towards the abdomen (3 points).

For glucose tolerance tests, after determination of overnight fasted blood glucose levels, mice were intraperitoneally injected with glucose at a dose of 2 g per kilogram of body weight [10% (w:v)]. Afterwards, blood glucose levels were determined from the blood of mouse tails at 15, 30, 60, 120, and 180 min using Contour next reactive glucose strips and a glucometer (Bayer).

For fecal microbiota transplantation (FMT), we proceeded as formerly described (52). In brief, mice fasted for 6 hours were orally gavaged for 3 consecutive days with 200 μl of an antibiotic cocktail consisting of neomycin (1 mg/ml) (Nzytech), ampicillin (1 mg/ml) (Nzytech), metronidazole (1 mg/ml) (Sigma), and vancomycin (0.5 mg/ml) (Sigma) in autoclaved water. The day afterwards, four to five fresh fecal pellets were pooled from donor mice in 600 μl of reduced buffer (0.5 mg/ml of cysteine and 0.2 mg/ml of Na_2_S in PBS) and vortexed for 1 min. Homogenates were then centrifuged at 500*g* for 5 min to remove large particles. Finally, 200 μl of fecal slurry was orally gavaged to recipient mice fasted for 4 hours twice a week for 2 weeks and then once a week until sacrifice. Following FMT, the remaining slurry was applied to the fur of recipient mice and their cages were replenished with fresh fecal pellets and dirty bedding from donor mice to ensure coprophagia.

For adoptive transfers of CD4 T cells, spleens and lymph nodes from 7-week-old Ly5.1 mice were harvested, dissociated through a 70-μm cell strainer, and resuspended in 5 ml of red blood cell lysis buffer (150 mM ammonium chloride, 10 mM sodium bicarbonate, and 100 µM EDTA, pH 7.4) for 5 min at 4°C. Then, cells were resuspended in 4 ml of MojoSort™ Buffer from MojoSort™ Mouse CD4 T Cell Isolation Kit (BioLegend) for total CD4 T cell isolation. Afterwards, cells were filtered again through a 70-μm cell strainer and resuspended in 2% fetal bovine serum (FBS) RPMI for counting. Total CD4 T cells were isolated following manufacturer’s instructions. Isolated cells were counted, checked for purity, and resuspended in sterile saline solution (0.9% NaCl). Finally, 6 × 10^6^ to 7 × 10^6^ CD4 T cells were injected into the retro-orbital sinus of isofluorane-anesthetized *Tfam*^fl/fl^*Cd4*^Cre^ mice.

For adoptive transfers of T_reg_ cells, 7-week-old Ly5.1 mice were pre-treated with IL-2. In brief, 1 µg of IL-2 (Peprotech, 212-12) was mixed with 5 µg of anti-IL-2 (Biolegend, 503706) per mouse in PBS and incubated at room temperature (RT) for 30 min. Mice were then intraperitoneally injected with 200 µl of the IL-2–anti-IL-2 complex solution for 3 consecutive days. On the fourth day, spleens and lymph nodes were harvested from pretreated mice and dissociated through a 70-μm cell strainer. Red blood cells were lysed as previously described. Cells were then resuspended in 4 ml of MojoSort™ Buffer from MojoSort™ Mouse CD4^+^CD25^+^ Regulatory T Cell Isolation Kit (BioLegend) for T_reg_ cell isolation. Cells were then filtered again through a 70-μm cell strainer and resuspended in 2% FBS RPMI for counting. CD25^+^ CD4 T cells were isolated following manufacturer’s instructions. Isolated cells were counted, checked for purity, and resuspended in sterile saline solution (0.9% NaCl). Finally, 2 × 10^6^ to 5 × 10^6^ T_reg_ cells were retro-orbitally injected into isofluorane-anesthetized *Tfam*^fl/fl^*Cd4*^Cre^ mice.

### Microbiota analysis by *16S* rRNA gene sequencing and qPCR

*16S* rRNA gene sequence analyses were performed as formerly reported (53). In brief, bacterial DNA from ileal, colonic, and fecal samples was extracted using the E.Z.N.A stool DNA kit (Omega Biotek) following manufacturer’s instructions. Amplicons of the V4 region of the *16S* rRNA gene were obtained in each sample using the following 5′-3′ primer pair: CCTACGGGAGGCAGCAG (FW) and ATTACCGCGGCTGCTGG (RV). Libraries were then sequenced using an Illumina MiSeq instrument, and sequences were curated and analyzed using the mothur (v.1.40.5) software package.(53)

For qPCR analysis of microbiota, 100 ng of bacterial DNA were included in the qPCR reaction, along with 5 μl of polymerase (Go Taq^®^ Master Mix, Promega) and 0.5 μl of each forward and reverse primer solution (5 μM stock) added to 384-well plates. The reaction was run on a Bio-Rad CFX 384 thermocycle. The 5′-3′ primer pairs (FW, RV. Sigma) used for qPCR are listed in table S1.

qPCR data were analyzed using the 2^−ΔΔCt^ method to calculate the relative abundance of bacteria members relative to *16S* rRNA gene, where ΔΔCt is the difference between the problem sample Ct values, and the control ones. After antibiotic treatment, fecal bacterial DNA concentrations were calculated by the amplification of the *16S* rRNA gene and using a standard curve of known bacterial DNA concentrations.

### Enzyme-linked immunosorbent assays (ELISA)

For LPS-binding protein (LBP) quantification, serum samples from mice were diluted and processed following manufacturer’s instructions (Enzyme Immunoassay for quantification of mouse LBP, Biometec). The plate was measured in a Dynex Opsys MR™ 96-plate reader (Aspect Scientific) at an excitation wavelength of 450 nm and an emission wavelength of 630 nm.

For serum IgA quantification, serum samples from mice were diluted 1:10,000 (v:v) following the manufacturer’s instructions (Mouse IgA ELISA Kit, Bethyl Laboratories). The plate was measured in a Dynex Opsys MR™ 96-plate reader (Aspect Scientific) at an excitation wavelength of 450 nm.

### Bacterial translocation analysis in the liver

A hepatic lobe was removed from culled mice under aseptic conditions, weighed, and homogenized in sterile-filtered 0.05% IGEPAL^®^ CA-630 (Sigma) and 1% bovine serum albumin (BSA) PBS solution using a Potter Elvehjem Tissue Homogenizer (Thomas Scientific). Then, Luria Bertani (LB) agar plates with no antibiotics were incubated with 100 μl of a 1:10 dilution of lysates and cultured for 96 hours in aerobic conditions at 37°C. Colonies were then enumerated and normalized to sample weight.

### Histological and immunofluorescence analysis of the intestine

Small intestine and colon were harvested by cutting below the stomach and above the cecum, and just below the cecum until the rectum, respectively. Fat was removed, and lumen was flushed with cold PBS to expel fecal and mucus content. Tissue samples were either fixed in 10% neutral buffered formalin for 24 hours and embedded in paraffin for histological analysis, or mounted in O.C.T Embedding Compound (Tissue-Tek, Sakura) and frozen in dry ice for their subsequent immunofluorescence staining.

Small intestine and colon sections were deparaffinized and stained as detailed in figure legends for their histological examination. Images were captured using the 5X objective of a vertical microscope AxioImager M1 (Zeiss) connected to a DMC6200 camera (Leica) and the LasX V4.13 software. Crypt depth and villus height were measured from the bottom of the crypt to the crypt–villus junction, and from there to the tip of the villus, respectively, using the NanoZoomer Digital Pathology software 2.7.25 version. Twenty-five to fifty crypt–villus units per mouse were quantified in a single-blind manner.

Colonic disease was scored as the sum of individual parameters for crypt loss and inflammation severity as well as the percentage of tissue damage, as previously described (42): (i) inflammatory cell infiltration: 0 = no evidence of inflammatory infiltrate; 1 = low level of cells infiltrating the tissue; 2 = thickening of lamina propria and clear infiltrating lymphocytes in epithelial tissue; and 3 = thickening of lamina propria and large boluses of inflammatory infiltrates; (ii) crypt loss: 0 = no evidence of crypt loss; 1 = mild crypt loss with a few areas affected; 2 = medium severity, greater crypt loss, and fewer crypts visible in large areas; 3 = large areas of total crypt loss and places where crypts are completely missing; and (iii) percentage of tissue damage: 0 = no area affected; 1 = 5 to 20% affected; 2 = 30 to 45% affected; 3 = 60 to 70% affected; and 4 = ≥ 80% affected. Seven to eight images per mouse were quantified in a single-blind manner.

Colon cryostat sections obtained from the O.C.T. molds were fixed in 4% paraformaldehyde (PFA), permeabilized in 0.1% Triton X-100 in PBS and then blocked in 2% BSA at RT. Sections were incubated either with rabbit anti-mouse ZO-1 (1:50 dilution, Invitrogen, 61-7300) or rabbit anti-mouse occludin (1:50 dilution, Invitrogen, 40-4700) at 4°C overnight. Slides were rinsed in PBS and then, incubated with Alexa Fluor™ 647–conjugated donkey anti-rabbit secondary antibody (1:500 dilution, Invitrogen, A-31573) and DAPI (1:10,000 dilution, Merck, 268298) for 1 hour at RT. Finally, slides were mounted with ProLong Reagent. Fluorescence images of colonic sections were acquired using the 40X objective of a LSM710 confocal microscope coupled with a vertical microscope AxioImager M2 (Zeiss) and the ZEN Black 2010 software. We used the following lasers and spectral detectors: DAPI (excitation: 405 nm; emission: 409 to 514 nm) and Alexa Fluor™ 647 (excitation: 633 nm; emission: 639 to 734 nm). Four to five images per section were quantified in a single-blind manner using the ImageJ software (v1.53n).

### RNA isolation, gene expression and qPCR analysis

Total RNA extraction was performed with a MagNA Lyser® homogenizer (Roche) using 1 cm of frozen tissue in 700 μl of TRIzol® reagent (Invitrogen). The RNA from the resulting aqueous phase was further purified using the RNeasy Mini Kit (QIAGEN). Quality and quantity were determined by measuring the absorbance at 260 and 280 nm on a Nanodrop One spectrophotometer (ThermoFisher).

cDNA libraries were prepared from total RNA and, then, validated and quantified by an Agilent 4150 Bioanalyzer in BGI Genomics or Agilent 4200 TapeStation in Haplox. After passing library inspection, stranded mRNA was sequenced either on the DNBSEQ™ platform or Illumina Novaseq Xplus, and FastQ files were generated containing nucleotide data and quality scores for each position. The quality of FastQ files was checked using FastQC (v0.11.9) RNA-sequencing reads were mapped to the *Mus musculus* reference genome GRCm39 using either Hisat2 (v2.2.1) or STAR (v2.5.2) software. Reads were then pre-processed with SAMtools v1.13 to transform Sequence Alignment/Map files into Binary Alignment/Map files and sorted. The number of reads covered by each gene was calculated by HTSeq-Count (v1.99.2).

Downstream data analysis was performed with R (v4.3.2). Differential gene expression (DEG) analysis was performed using DESeq2 (v1.44). Genes with *P* value < 0.05 and |log_2_-fold change| > 0.6 were determined to show statistically significant differences in group comparison. Over-representation analysis (ORA) and gene set enrichment analysis (GSEA) were performed using clusterProfiler (v4.8.3) package in GO, KEGG, WikiPathways, Reactome and the Hallmarks of the Molecular signatures databases. PCA plots, chord diagrams and heatmaps were visualized by using ggplot2 (v3.4.4), circlize (v0.4.15) and pheatmap (v1.0.12), respectively.

For qPCR analysis, reverse transcription was performed using 500 ng of RNA extracts and the Maxima™ First Strand cDNA Synthesis Kit and dsDNase (ThermoFisher). The reaction was performed in a ProFlex™ PCR System thermocycler (Applied Biosystems) at 25°C for 10 min, followed by 50°C for 15 min, and 85°C for 5 min. cDNA samples were then cooled to 4°C and stored at −20°C for qPCR analysis. Amplification conditions were determined by the primers to present amplification efficiency close to 100% and a single peak in melt-curve analyses. A 1:25 dilution of cDNA samples was included in the qPCR reaction along with 5 μl of polymerase (Go Taq^®^ Master Mix, Promega) and 0.5 μl of each forward and reverse primer solution (5 μM stock) added to 384-well plates. The reaction was run on a Bio-Rad CFX 384 thermocycle. The 5′-3′ primer pairs (FW, RV. Sigma) used for qPCR analysis are listed in table S1.

qPCR data were analyzed using the 2^−ΔΔCt^ method to calculate the relative changes in gene expression, where ΔΔCt is the difference between the problem sample Ct values, and the control ones. The relative expression in each figure represents the induction levels of the gene of interest relative to *Hprt* and *Pp1a* in ileal or colonic samples or relative to *B2m* in liver samples.

### Short-chain fatty acid (SCFA) quantification

Standards of eight straight and branched-chain SCFAs [acetic acid (AA), propionic acid (PA), isobutyric acid (i-BA), butyric acid (BA), 2-methylbutyric acid (2-Me-BA), isovaleric acid (i-VA), valeric acid (VA), and 3-methylvaleric acid (3-Me-VA)], 3-nitrophenylhydrazine (3NPH), N-(3-dimethylaminopropyl)-N′-ethylcarbodiimide hydrochloride (EDC), formic acid, and pyridine were purchased from Sigma-Aldrich. Acetonitrile (ACN) was purchased from VWR. Calibration curves were constructed using a mixed SCFA solution in ACN-water (50:50, v/v), with ranges of 1 to 10,000 µM for AA and 0.1 to 1000 µM for PA, i-BA, BA, 2-Me-BA, i-VA, and VA.

SCFAs were extracted from 50 mg of feces using 100 µl of ACN-water (50:50, v/v) containing 5 µM internal standard, homogenized with a FastPrep-24 5G system (MP Biomedicals), incubated at 800 rpm for 15 min at 10°C, and centrifuged at 14,000*g* for 30 min at 4°C. Forty microliters of the supernatant was subsequently mixed with 20 µl of 200 mM 3NPH and 120 mM EDC/6% pyridine in ACN-water (50:50, v/v) and incubated for 30 min at 40°C. After cooling to RT, samples were diluted 20-fold with 10% ACN in water, centrifuged at 14,000*g* for 10 min at 4°C, and analyzed by UPLC-MS/MS. Standards and blanks were processed using the same derivatization protocol.

SCFA analysis was conducted following a previously described protocol (54). Analyses were carried out using an Agilent 1260 Infinity II system coupled to an Ultivo 6465 Triple Quadrupole LC-MS, equipped with an Agilent Jet Stream ESI source and controlled via MassHunter Workstation (Agilent Technologies). Multiple reaction monitoring (MRM) acquisition was conducted using the optimal transitions and parameters listed in table S2.

### Luminex detection of cytokines

Blood (100 to 120 μl) was collected in BD Microtainer^®^ tubes from the facial vein or after culling mice and centrifuged at 6000*g* for 10 min at 4°C to obtain the serum fraction. Cytokines from serum samples were quantified using the multiplexed bead-based immunoassay Cytokine & Chemokine 26-Plex Mouse ProcartaPlex™ Panel 1 (Invitrogen, EPX260-26088-901) following the manufacturer’s instructions. Measurements under the detection limit were considered zero.

### Quantification of senescence-associated β-galactosidase activity

Tissues were lysed in T-PER Tissue Protein Extraction Reagent (Thermo Scientific, 78510) using a MagNA Lyser^®^ homogenizer (Roche). Lysates were then centrifuged at 10,000*g* for 5 min and the supernatant was collected. Fifty microliters of protein lysates were mixed with 50 μl of β-galactosidase Assay Reagent (Thermo Scientific, 75705) for the assay. The reaction was incubated for 30 min and the absorbance was finally measured at 405 nm. Values were normalized to total protein levels.

### Flow cytometry

After blood was extracted in vivo from the facial vein in 1.5-ml tubes containing 0.5 M EDTA, 200 μl was immediately transferred into a 15-ml tube containing 1 mM EDTA PBS. Samples were centrifuged at 400*g* for 5 min at 4°C and erythrocytes were removed using red blood cell lysis buffer twice. After washing with 1 mM EDTA PBS, cells pellets were finally resuspended in 1 ml of 2% FBS RPMI until cells were stained.

Mice were euthanized using CO_2_ narcosis followed by perfusion with cold PBS. Peyer’s patches were harvested from the small intestine and dissociated through a premoistened 70-μm cell strainer. Cell suspension was centrifuged at 400*g* for 5 min at 4°C. Finally, cell pellets were resuspended in 1 ml of 2% FBS RPMI for counting.

Lymphoid cells from the colonic lamina propria were isolated as previously described (55). In brief, colon samples between the cecum and rectum were obtained and cleaned from fat and feces. Tissues were cut longitudinally and washed with cold PBS. Then, tissues were cut transversely into 1 cm–long fragments and mixed in prewarmed PBS with 5 mM EDTA, 14 mM HEPES, and 10% FBS under shaking conditions at 180 rpm for 30 min at 37°C. After washing with PBS, tissue pieces were then minced and mixed in prewarmed RPMI with 25 mM HEPES, 10% FBS, and 300 U/ml of collagenase type VIII (Sigma, C2139) under shaking conditions at 180 rpm for 45 min at 37°C. Digested tissue was filtered through a premoistened 70-μm cell strainer, washed with PBS with 5 mM EDTA, 14 mM HEPES, and 10% FBS, and centrifuged at 650*g* for 5 min at RT. To further enrich in lymphocytes, supernatants were centrifuged in a 40%–70% Percoll gradient (Sigma, GE17-0891-01) at 750*g* for 20 min at RT without acceleration or brake. Isolated cells were then washed with PBS and resuspended in 2% FBS RPMI for counting.

For surface staining, ≤ 2 × 10^6^ cells were incubated with anti-CD16/32 (BD Pharmingen, 553142) and either the Zombie NIR™ Fixable Viability Kit (BioLegend, 423106), the Zombie Yellow™ Fixable Viability Kit (BioLegend, 423104) or the Ghost Dye™ Violet 540 (Tonbo Biosciences, 525 13-0879) live/dead cell marker for 15 min at 4°C before staining. Then, surface staining was performed using fluorochrome-conjugated antibodies in Brilliant Stain Buffer (BD Bioscience, 566349) for 30 min at 4°C. The antibodies used for surface antigen staining are listed in table S3.

To detect cytokine production ex vivo, cell suspensions were resuspended at ≤ 10^6^ cells/ml in RPMI containing 10% FBS and stimulated with 50 ng/ml of phorbol 12-myristate 13-acetate (PMA, Sigma, P1585) and 1 µg/ml of ionomycin (Tocris, 1704) in the presence of brefeldin A (BD GolgiPlug, BD Bioscience, 555029) for 4 hours at 5% CO_2_ and 37°C. Cells were then processed for surface marker staining as described above. For intracellular cytokine and transcription factor staining, cells were fixed in Foxp3 / Transcription Factor Staining Buffer (eBioscience, 00–5523-00) for 20 min at RT, then washed with its Permeabilization buffer. Unstimulated cells and Fluorescence Minus One (FMO) controls were utilized to evaluate stimulation and intracellular staining procedures, respectively. The antibodies used for intracellular antigen staining are listed in table S3.

Flow cytometry experiments were acquired using a 4-Laser (V/B/YG/R) Aurora flow cytometer and the Spectroflo software (Cytek Biosciences), with compensation beads as controls. Subsequently, multiparametric analysis was performed utilizing the OMIQ platform (www.omiq.ai) after preliminary cleaning of debris, dead cells and aggregates. First, the flowCut algorithm was run to remove anomaly acquired events in all files analyzed. Then, CD4 T cells were selected and subsampled ≤ 10,000 events, followed by dimension reduction and clustering using unsupervised uniform manifold approximation and projection (UMAP) and ClusterX algorithms, respectively, to visualize the different T cell subsets in groups. Finally, results were plotted and identified depending on their mean fluorescence marker expression.

### Determination of IgA-coating of fecal bacteria

Fecal pellets were weighed and incubated in 1 ml of filtered PBS per 100 mg of feces for 20 min at 4°C. Then, samples were homogenized vortexing at full speed for 1 min and centrifuged at 500*g* for 5 min at 4°C. One hundred microliters of supernatant were transferred to a fresh tube, washed in 1 ml of blocking buffer (filtered 2% BSA in PBS) and centrifuged at 10,000*g* for 5 min at 4°C to pellet bacteria. Bacteria were further incubated in 100 μl of blocking buffer for 15 min at 4°C, washed, and stained with PE-conjugated anti-IgA (1:100 dilution, eBioscience, 12-4204-82, mA-6E1 clone) in blocking buffer for 45 min at 4°C. After washing, stained bacteria were fixed in 100 μl of 4% PFA overnight at 4°C. The next day, bacteria were washed twice and then stained with 100 μl of Hoechst (Dilution 1/20, Invitrogen, D1306) in blocking buffer containing 0.01% Tween and 1 mM EDTA and immediately acquired in a FACSCanto II Cytometer (BD) with the SSC threshold set to 200.

### Statistical analyses and figure design

Statistical analyses were performed using GraphPad Prism 9 or Past 3.22 software. Outliers were identified and excluded by the ROUT method (5%). If data followed a normal distribution after applying the Shapiro–Wilk test, comparisons between two datasets were performed using the unpaired two-tailed Student’s *t* test, and comparisons between more than two datasets were performed using the one or two-way analysis of variance (ANOVA) or mixed-effects analysis with Tukey’s or Šidák’s multiple comparison tests. If not, comparisons between two or more datasets were calculated using the non-parametric Mann–Whitney *U* test and Kruskal–Wallis *H* test with Dunn’s multiple comparison test, respectively. For categorical variables, Fisher’s exact test was used. For survival curves, the log-rank Mantel–Cox test was applied. Permutational multivariate analysis of variance (PERMANOVA) was performed based on 9999 permutations. Differences with *P* values ≤ 0.05 were considered significant. *P ≤ 0.05; **P ≤ 0.01; ***P ≤ 0.001; and ****P ≤ 0.0001. Unless otherwise stated, experimental data were represented as means ± the standard error of the mean (SEM), where each dot was an individual biological sample of each experimental group. In the figure legends, (*n*) denotes the number of mice per group per experiment and (*N*) the number of independent experiments performed. Box-and-whisker plots represent the interquartile range between the first and third quartiles (25th and 75th percentiles, respectively), the median, and the maximal and minimal values. Violin plots represent the interquartile range between the first and third quartiles (25th and 75th percentiles, respectively) and the median. Figures were designed using GraphPad Prism 9 and Adobe Illustrator (v29.0.1).

## Supplementary Material

Supplementary Material

## Data Availability

Colon RNA-sequencing raw data after the CD4 T cell and T_reg_ cell–enriched adoptive transfer strategies, as well as microbiota *16S rRNA* gene sequencing raw data are publicly available via NCBI with BioProject numbers PRJNA1105872, PRJNA1185212, and PRJNA1107518, respectively. Tabulated data underlying the figures are provided in data file S1. All other data associated with this study are available either in the main text or the supplementary materials.

## One-Sentence Summary

T_reg_ cells safeguard the intestinal barrier to prevent inflammaging and senescence in a mouse model of premature aging.
