# Supplementary material for: CD4 T cell therapy counteracts inflammaging and senescence by preserving gut barrier integrity
Source: Sci Immunol. Author manuscript; Available in PMC 2025 Oct 6. (PMC7618201; doi:10.1126/sciimmunol.adv0985)
Supplement: Supplementary Material [file EMS209263-supplement-Supplementary_Material.pdf]

Supplementary Materials for

Treg therapy counteracts inflammaging and senescence by preserving gut barrier integrity

Manuel M. Gómez de las Heras, Elisa Carrasco, Mario Pérez-Manrique, Naohiro Inohara, Sandra Delgado-Pulido, Álvaro Fernández-Almeida, María I. Gálvez-Castaño, Isaac Francos-Quijorna, Carolina Simó, Virginia García-Cañas, J. Ignacio Escrig-Larena, Juan Francisco Aranda, Gonzalo Soto-Heredero, Enrique Gabandé-Rodríguez, Eva María Blanco, Joyce Días-Almeida, Gabriel Núñez, and María Mittelbrunn

Corresponding author: mmittelbrunn@cbm.csic.es

The PDF file includes:

Figs. S1 to S11.
Tables S1 to S3.
Captions for data file S1.

Other Supplementary Materials for this manuscript include the following:

Data file S1.
MDAR Reproducibility Checklist.

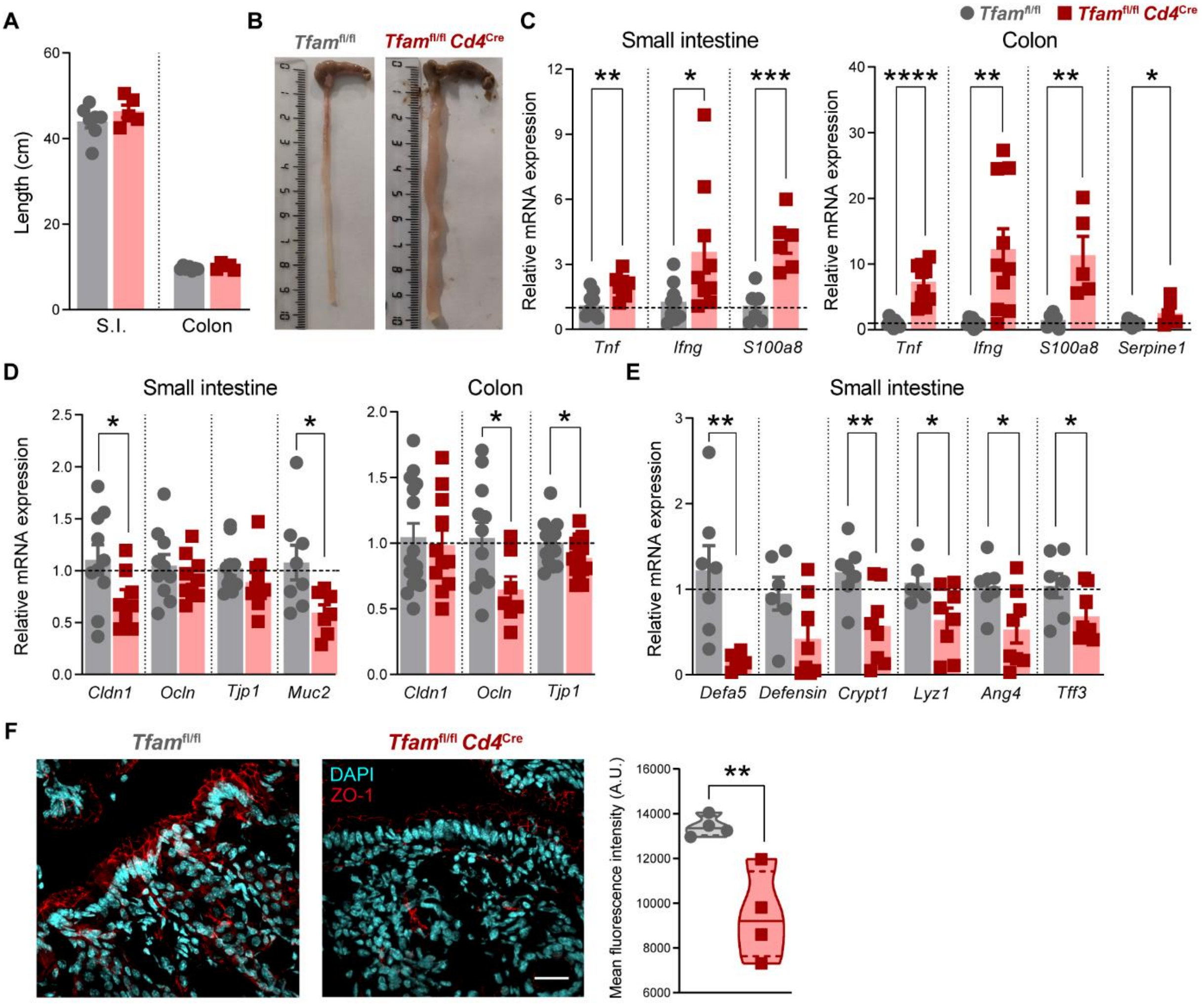

Fig. S1. The intestine of *Tfam*$^{fl/fl}$*Cd4*^{Cre} mice display a strong inflammatory signature with altered cell-to-cell junctions. (**A**) Small intestine (S.I.) and colon length of 12-month-old *Tfam*$^{fl/fl}$ and *Tfam*$^{fl/fl}$*Cd4*^{Cre} mice (n = 5 to 7). (**B**) Representative images of *Tfam*$^{fl/fl}$ and *Tfam*$^{fl/fl}$*Cd4*^{Cre} colons. (**C**) Bar plots showing relative mRNA levels of genes associated with inflammation (*Tnf*, *Infg*, and *S100a8*) in the small intestine and the colon, and with colon fibrosis (*Serpine1*) (n = 3 to 6). (**D**) Bar plots showing relative mRNA levels of genes encoding proteins involved in tight junctions (*Cldn1*, *Ocln*, and *Tjp1*) and mucin-2 (*Muc2*) in the small intestine and the colon (n = 3 to 6). (**E**) Bar plots showing relative mRNA levels of genes encoding antimicrobial peptides (*Defa5*, *Defensin*, *Crypt1*, *Lyz1*, *Ang4* and *Tff3*) in the small intestine (n = 3 to 4). (**F**) Representative image (scale bar: 20 µm) and quantification of ZO-1 immunofluorescence staining in the colonic epithelium (n = 4). Data are (A and F) representative of N = 2 or (C to E) pooled from N = 2 to 4 independent experiments. Data are shown as means ± SEM, where each dot is a biological sample. P values were determined by unpaired Student's t test. *$P \leq 0.05$; **$P \leq 0.01$; ***$P \leq 0.001$; and ****$P \leq 0.0001$.

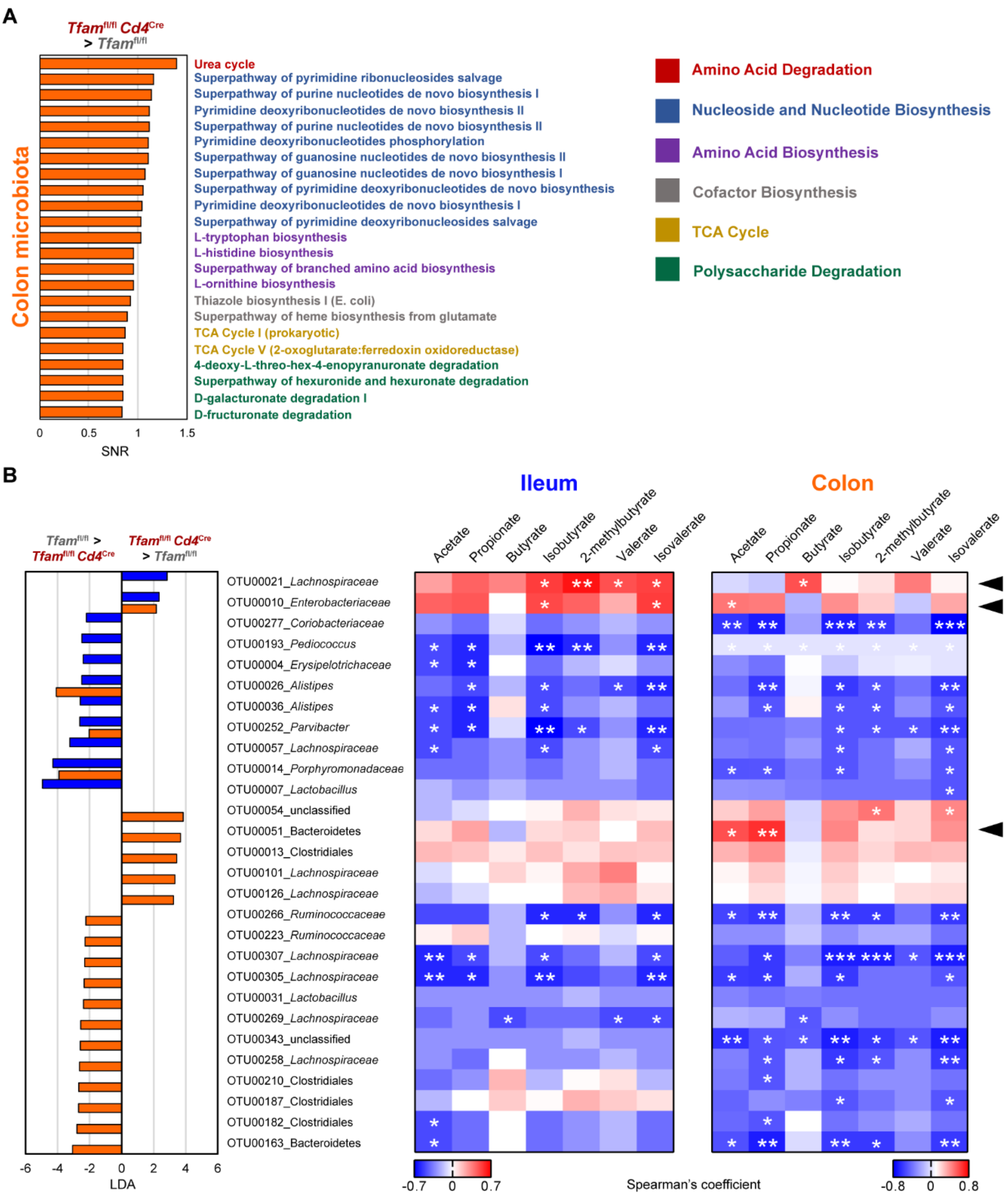

Fig. S2. Predictive metabolic profiling of the gut microbiota in *Tfam*$^{fl/fl}$*Cd4*^{Cre} mice. (**A**) PICRUSt2 prediction of metabolic pathways in colon-resident microbiota metagenomic data from 12-month-old *Tfam*$^{fl/fl}$*Cd4*^{Cre} versus *Tfam*$^{fl/fl}$ mice. SNR: signal-to-noise ratio; TCA: tricarboxylic acid cycle. (**B**) Left: differentially abundant OTUs depicted with lineal discriminant analysis (LDA) values of linear discriminant effect size (LEfSe, $P < 0.05$; false discovery rate, $q < 0.05$; fold change > 5; maximal abundance > 0.001) comparing ileal and colonic microbiota in *Tfam*$^{fl/fl}$*Cd4*^{Cre} versus *Tfam*$^{fl/fl}$ mice. Right: heatmap depicting Spearman's rank correlation coefficients from the correlation analysis between differentially abundant OTUs in ileum and colon, and the concentration of short-chain fatty acids. P values were determined using the OTU association command of mothur (v.1.40.5) software package. *$P \leq 0.05$; **$P \leq 0.01$; and ***$P \leq 0.001$.

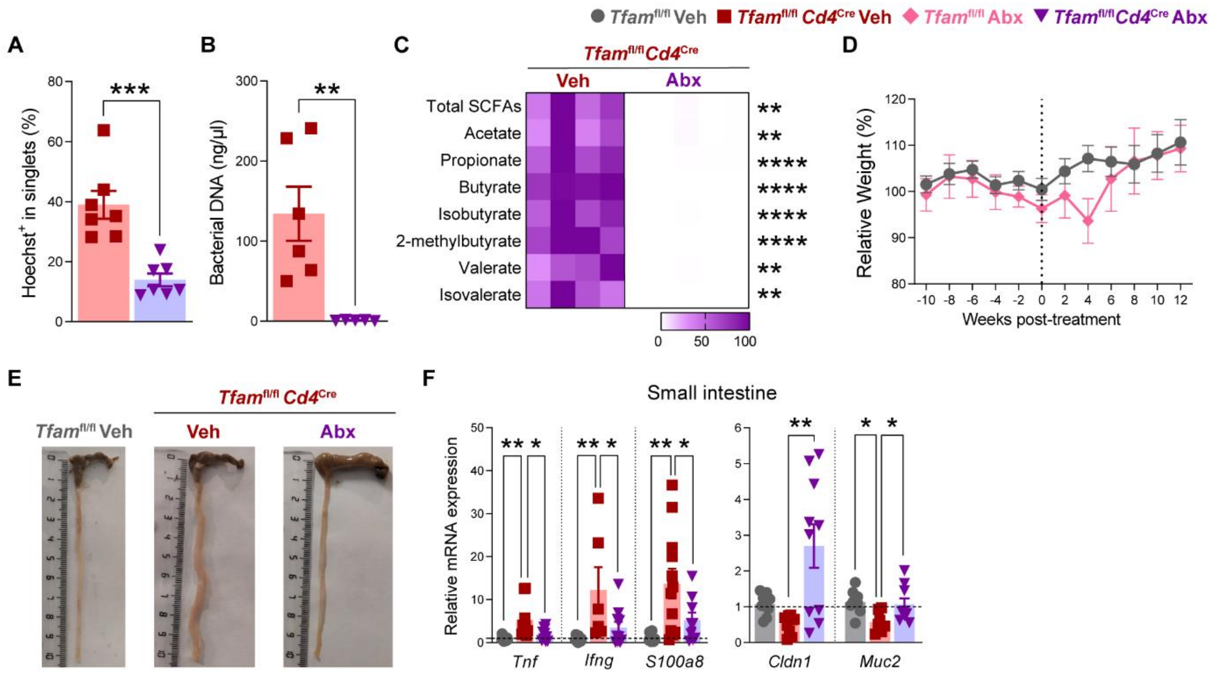

Fig. S3. Effects of microbiota depletion in *Tfam*$^{fl/fl}$*Cd4*^{Cre} mice. (**A**) Flow cytometry quantification of bacteria in fecal samples from 12-month-old *Tfam*$^{fl/fl}$*Cd4*^{Cre} mice treated with antibiotics (Abx) or vehicle (Veh) for 8 weeks (n = 3 to 4). (**B**) qPCR quantification of *16S* rRNA gene in bacterial DNA from the feces (n = 5 to 6). (**C**) Heatmap portraying normalized values of microbiota-derived short-chain fatty acids (SCFAs) in the feces (n = 4 to 5). (**D**) Body weights of *Tfam*$^{fl/fl}$ mice treated with Abx or Veh relative to the start of treatment (n = 3 to 8). (**E**) Representative images of colons from *Tfam*$^{fl/fl}$ mice treated with Veh and *Tfam*$^{fl/fl}$*Cd4*^{Cre} mice treated with Abx or Veh. (**F**) Bar plots showing relative mRNA levels of genes associated with inflammation (*Tnf*, *Infg*, and *S100a8*), with tight junctions (*Cldn1*) and mucus (*Muc2*) in the small intestine (n = 3 to 5). Data are (A and F) pooled from N = 2 or (C to E) representative of N = 2 independent experiments. Data are shown as means ± SEM, where each dot is a biological sample. P values were determined by (A to C) unpaired Student's t test, (D) two-way analysis of variance (ANOVA) with Tukey's multiple comparisons test, or (F) one-way ANOVA with Tukey's multiple comparisons test. *$P \leq 0.05$; **$P \leq 0.01$; ***$P \leq 0.001$; and ****$P \leq 0.0001$.

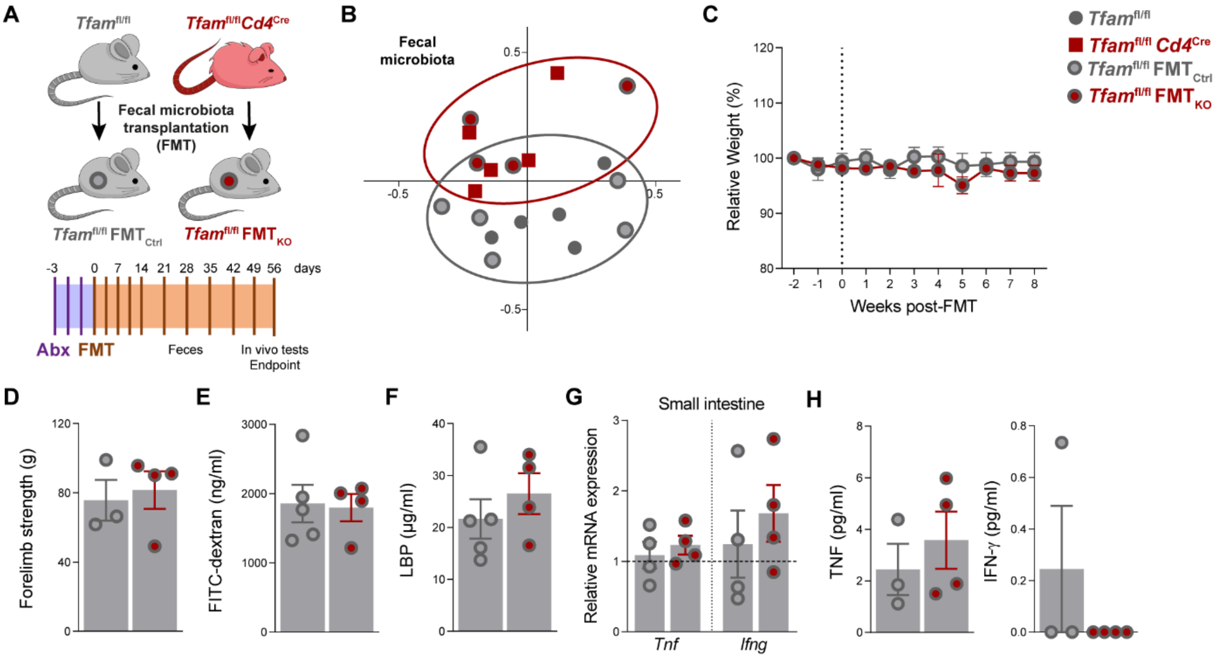

Fig. S4. Fecal microbiota transplantation from *Tfam*$^{fl/fl}$*Cd4*^{Cre} mice into young control *Tfam*$^{fl/fl}$ mice. (**A**) Experimental design of fecal microbiota transplantation (FMT) from 12-month-old *Tfam*$^{fl/fl}$ and *Tfam*$^{fl/fl}$*Cd4*^{Cre} mice into 4-month-old *Tfam*$^{fl/fl}$ mice, denoted as *Tfam*$^{fl/fl}$FMT$_{Ctrl}$ and *Tfam*$^{fl/fl}$FMT$_{KO}$ mice, respectively. (**B**) Nonmetric multidimensional scaling (NMDS) plots showing β-diversity values (θYC indexes) of fecal microbiota 28 days after the first FMT (n = 4 to 5). (**C**) Body weights of recipient mice relative to the beginning of the treatment (n = 5). (**D**) Grip strength analysis (n = 3 to 4). (**E**) Concentration of FITC-dextran in the serum (n = 4 to 5). (**F**) Levels of LPS-binding protein (LBP) in the serum (n = 4 to 5). (**G**) Bar plots showing relative mRNA levels of genes encoding TNF and IFN-γ in the small intestine (n = 4). (**H**) Concentration of the proinflammatory cytokines TNF and IFN-γ in the serum (n = 3 to 4). (B to H) Data are representative of N = 2 independent experiments. Data are shown as means ± SEM, where each dot is a biological sample. P values were determined by (B) permutational multivariate analysis of variance (PERMANOVA), (C) two-way analysis of variance (ANOVA) with Šídák's multiple comparisons test, or (D to H) unpaired Student's t test.

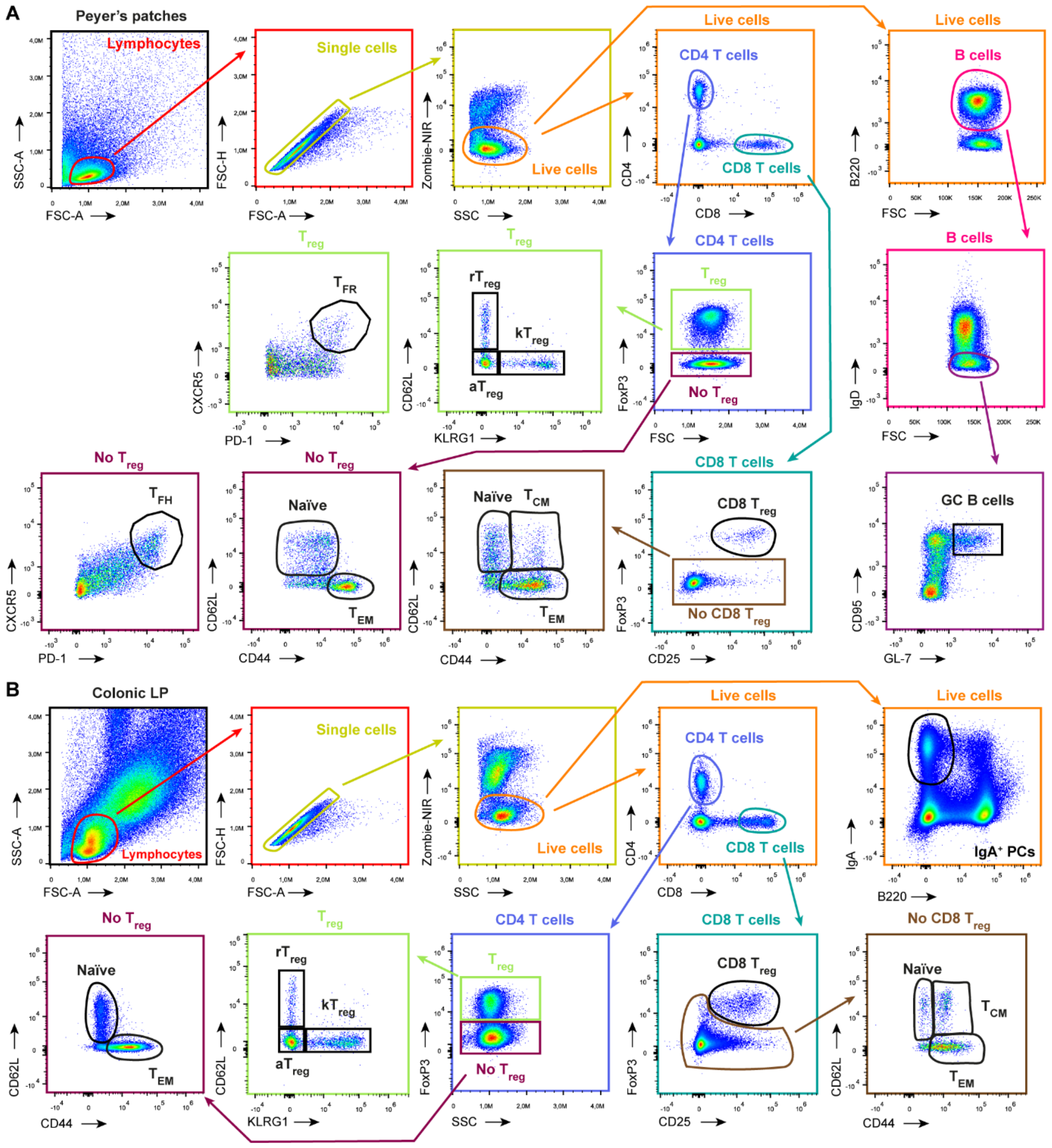

Fig. S5. Gating strategy to identify immune cell subpopulations in the mouse intestine. (**A**) Gating strategy to identify CD4 and CD8 T cell subsets and germinal center (GC) B cells in Peyer's patches. (**B**) Gating strategy followed to identify CD4 and CD8 T cell subsets and IgA-producing plasma cells (PCs) in the colonic lamina propria (LP).

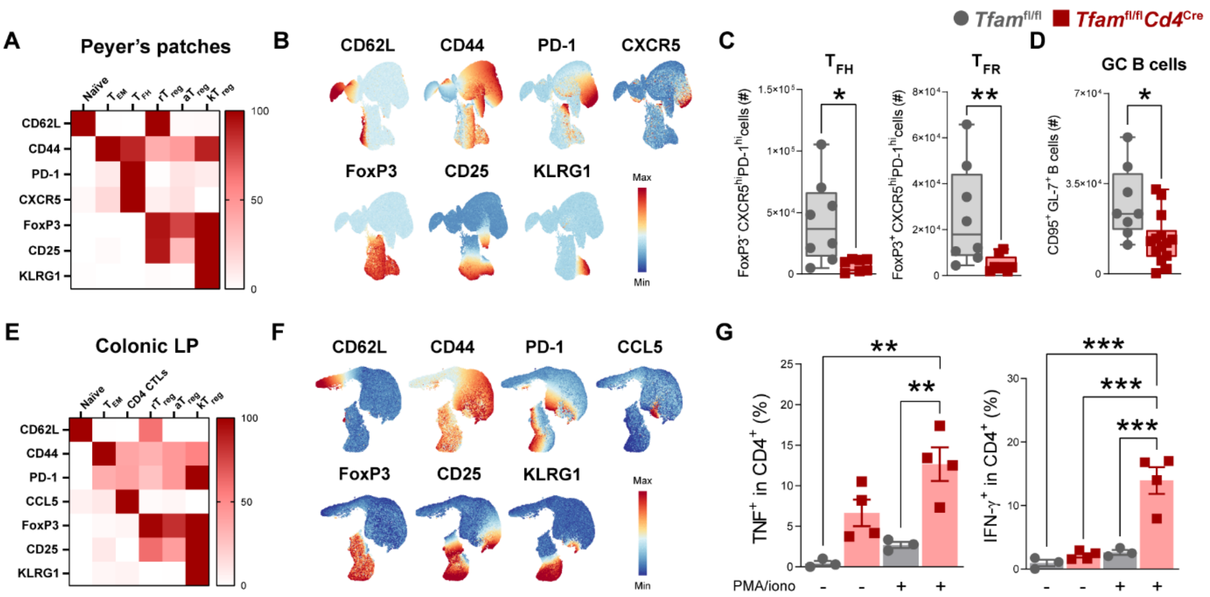

Fig. S6. $Tfam^{fl/fl}Cd4^{Cre}$ mice show deficient germinal centers and a proinflammatory CD4 T cell compartment in the intestine. (**A**) Heatmap depicting normalized values of mean fluorescence intensity of each marker in every CD4 T cell cluster in Peyer's patches (PPs) of 12-month-old $Tfam^{fl/fl}$ and $Tfam^{fl/fl}Cd4^{Cre}$ mice. (**B**) UMAP representation of the expression levels of markers used to identify CD4 T cell clusters in PPs. (**C**) Absolute numbers of T follicular helper (T_{FH}) and T follicular regulatory (T_{FR}) cells in PPs (n = 3 to 5). (**D**) Absolute numbers of germinal center (GC) B cells in PPs (n = 3 to 5). (**E**) Heatmap depicting normalized values of mean fluorescence intensity of each marker in every CD4 T cell cluster in the colonic lamina propria (cLP). (**F**) UMAP representation of the expression levels of markers used to identify CD4 T cell clusters in the cLP. (**G**) Quantification of TNF- and IFN-γ–producing cLP CD4 T cells stimulated ex vivo (+) or not (−) with phorbol 12-myristate 13-acetate (PMA)–ionomycin (iono) in the presence of brefeldin A for 4 hours (n = 3 to 4). Data are (C and D) pooled from N = 2 to 3 or (G) representative of N = 2 to 3 independent experiments. Data are shown as means ± SEM, where each dot is a biological sample. P values were determined by (C and D) unpaired Student's t test, (C) two-tailed Mann–Whitney U test (T_{FR}), or (G) one-way analysis of variance (ANOVA) with Tukey's multiple comparisons test. $*P \leq 0.05$; $**P \leq 0.01$; and $***P \leq 0.001$.

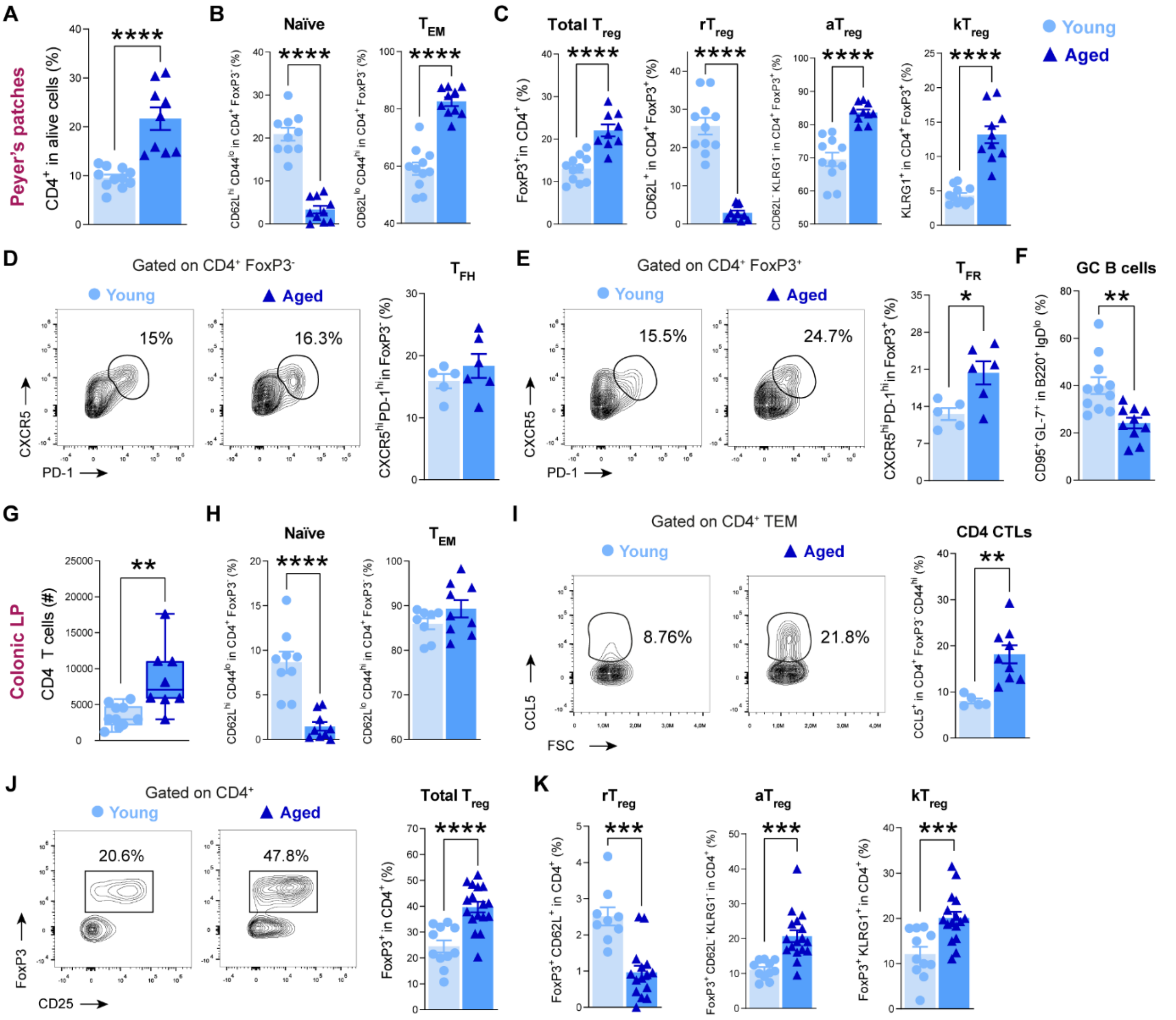

Fig. S7. Characterization of CD4 T cell subsets in Peyer's patches and the colonic lamina propria of naturally aged wild-type mice. (**A**) Percentage of CD4 cells in Peyer's patches (PPs) of young (3-month-old) and aged (24-month-old) wild-type mice (*n* = 4 to 6). (**B**) Quantification of naïve and effector/memory-like (T_{EM}) CD4 T cells in PPs (*n* = 4 to 6). (**C**) Quantification of total, resting (r), activated (a), and KLRG1$^+$ (k) regulatory T cells (Tregs) in PPs (*n* = 4 to 6). (**D** and **E**) Representative contour plots and quantification of (D) T follicular helper (T_{FH}) and (E) T follicular regulatory (T_{FR}) cells in PPs (*n* = 5 to 6). (**F**) Quantification of germinal center (GC) B cells in PPs (*n* = 4 to 6). (**G**) Absolute numbers of CD4 T cells in the colonic lamina propria (cLP) (*n* = 4 to 5). (**H**) Quantification of naïve and T_{EM} CD4 T cells in the cLP (*n* = 4 to 5). (**I**) Representative contour plots and quantification of CD4 cytotoxic T lymphocytes (CTLs) in the cLP (*n* = 4 to 5). (**J**) Representative contour plots and quantification of total cLP Tregs (*n* = 5 to 6). (**K**) Quantification of rTregs, aTregs, and kTregs in the cLP (*n* = 5 to 6). Data are (A to C, and F to K) pooled from *N* = 2 to 3 or (D and E) representative of *N* = 2 independent experiments. Data are shown as means ± SEM, where each dot is a biological sample. *P* values were determined by unpaired Student's *t* test. *$P \leq 0.05$; **$P \leq 0.01$; ***$P \leq 0.001$; and **** $P \leq 0.0001$.

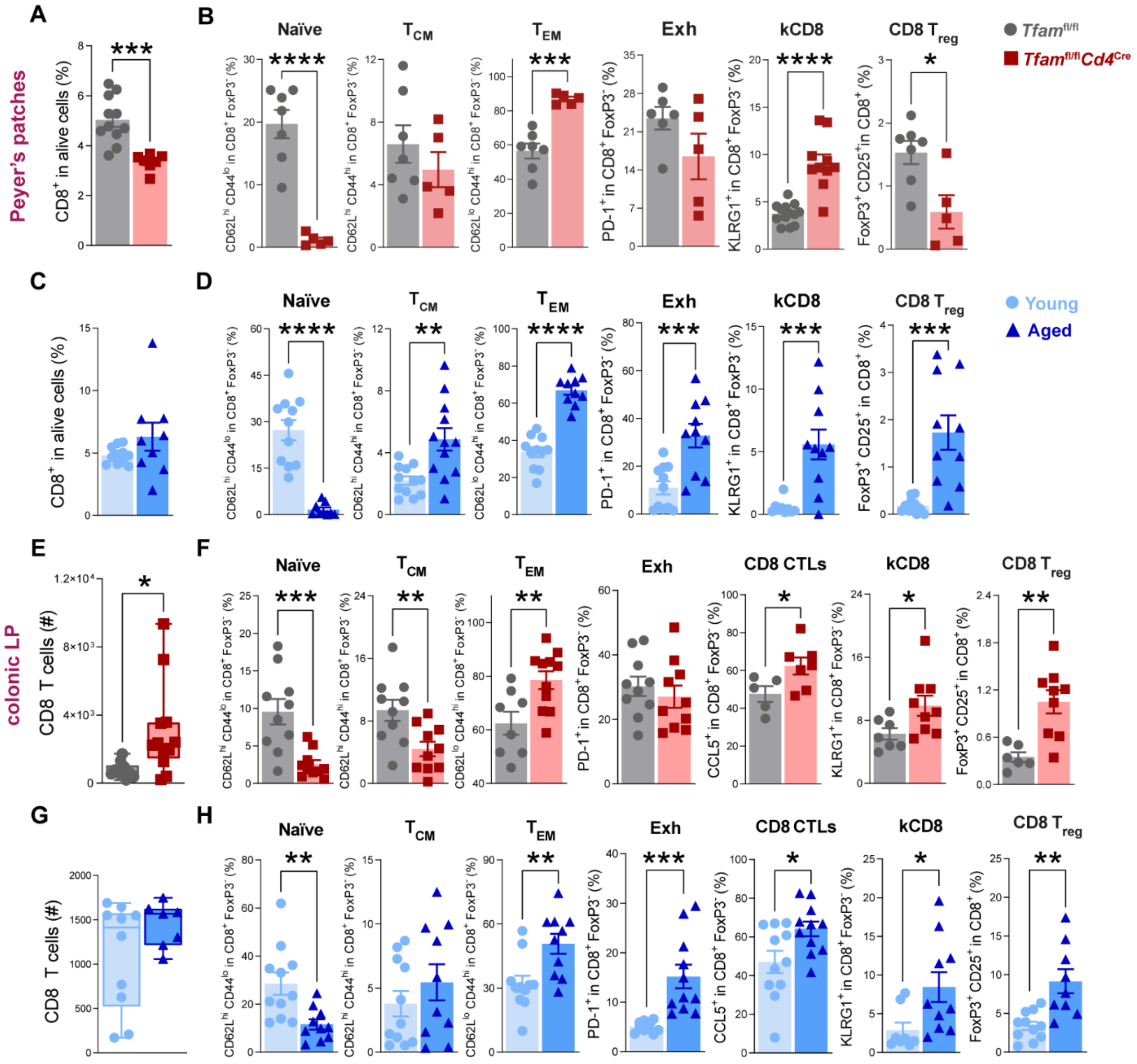

Fig. S8. Analysis of CD8 T cells in the intestine of *Tfam*$^{fl/fl}$*Cd4*^{Cre} mice compared to naturally aged wild-type mice. (**A** and **C**) Percentage of CD8 T cells in Peyer's patches (PPs) of (A) 12-month-old *Tfam*$^{fl/fl}$ and *Tfam*$^{fl/fl}$*Cd4*^{Cre} mice (n = 5 to 6), and (C) young (3-month-old) and aged (24-month-old) wild-type mice (n = 4 to 6). (**B** and **D**) Quantification of naïve, central memory (T_{CM}), effector/memory-like (T_{EM}), exhausted (Exh), KLRG1^{+} (kCD8), and regulatory (CD8 Treg) CD8 T cell subsets in PPs of (B) adult *Tfam*$^{fl/fl}$*Cd4*^{Cre} mice (n = 3 to 7), and (D) aged wild-type mice (n = 4 to 6) compared to their respective controls. (**E** and **G**) Absolute number of CD8 T cells in the colonic lamina propria (cLP) of (E) adult *Tfam*$^{fl/fl}$*Cd4*^{Cre} mice (n = 4 to7), and (G) aged wild-type mice (n = 5 to 6) compared to their respective controls. (**F** and **H**) Quantification of naïve, T_{CM}, T_{EM}, Exh, cytotoxic T lymphocytes (CD8 CTLs), kCD8, and CD8 Tregs in the cLP of (F) adult *Tfam*$^{fl/fl}$*Cd4*^{Cre} mice (n = 4 to 6) and (H) aged wild-type mice (n = 5 to 6 mice) compared to their respective controls. Data are pooled from N = 2 to 3 independent experiments. Data are shown as means ± SEM, where each dot is a biological sample. P values were determined by unpaired Student's t test. *$P \leq 0.05$; **$P \leq 0.01$; ***$P \leq 0.001$; and **** $P \leq 0.0001$.

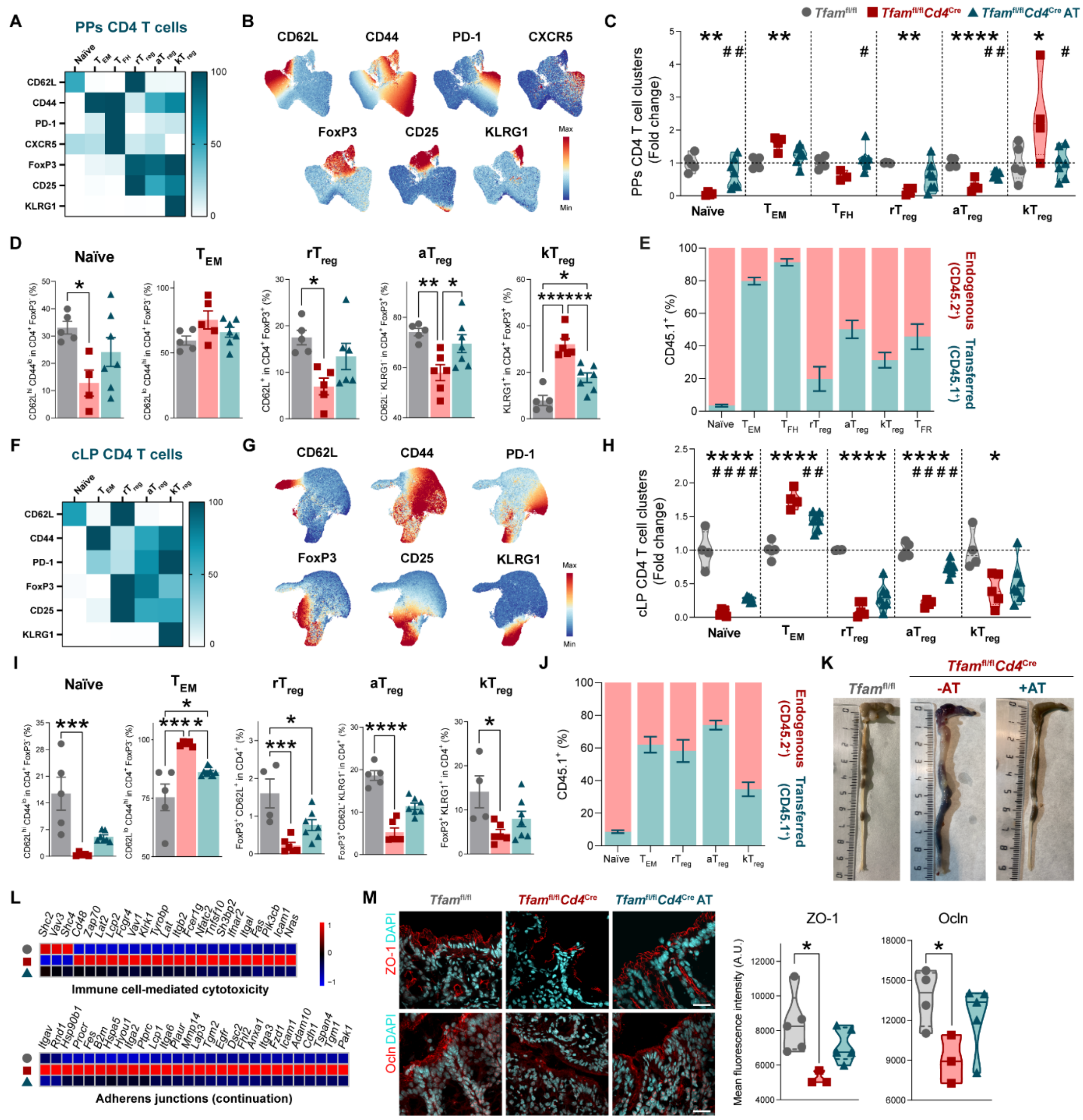

Fig. S9. CD4 T cell therapy recovers gut immune homeostasis strengthening gut barrier integrity in *Tfam*$^{fl/fl}$*Cd4*^{Cre} mice. (**A**) Heatmap portraying normalized values of mean fluorescence intensity of each marker in CD4 T cell clusters in Peyer's patches (PPs) of 10-month-old *Tfam*$^{fl/fl}$ and *Tfam*$^{fl/fl}$*Cd4*^{Cre} mice after the adoptive transfer of CD4 T cells (AT). (**B**) UMAP representation of the expression levels of markers used to identify PP subsets. (**C**) Fold change of PP clusters compared to control mice (n = 3 to 7). (**D**) Quantification of naïve, effector/memory-like (T_{EM}), resting (r), activated (a), and KLRG1$^+$ (k) regulatory T cells (Tregs) in PPs (n = 4 to 7). (**E**) Mean chimeric ratio of PP CD4 T cell subsets. (**F**) Heatmap portraying normalized values of mean fluorescence intensity of each marker in CD4 T cell clusters in the colonic lamina propria (cLP). (**G**) UMAP representation of the expression levels of markers used to identify cLP clusters. (**H**) Fold change of cLP clusters compared to control mice (n = 4 to 7). (**I**) Quantification of cLP CD4 T cell subsets (n = 5 to 7). (**J**) Mean chimeric ratio of cLP CD4 T cell subsets. (**K**) Representative images of the colon. (**L**) Heatmap depicting expression of genes in the colon RNA-sequencing analysis. (**M**) Representative image (scale bar: 20 μm) and quantification of ZO-1 and occludin (Ocln) immunofluorescence staining in

the colon (n = 3 to 5). Data are representative of N = 2 independent experiments. Data are shown as means ± SEM, where each dot is a biological sample. P values were determined by (C, D, H, and I) one-way analysis of variance (ANOVA) with Tukey's multiple comparisons test. (I and M) P values were determined by (T_{EM}, rTregs, kTregs, and Ocln) one-way ANOVA or (naïve, aTregs, and ZO-1) Kruskal–Wallis H test with Dunn's multiple comparison test. $Tfam^{fl/fl}$ versus $Tfam^{fl/fl}Cd4^{Cre}$ (*); $Tfam^{fl/fl}Cd4^{Cre}$ versus $Tfam^{fl/fl}Cd4^{Cre}$ AT ($^{\#}$). $^{*,\#}P \leq 0.05$; $^{**,\#\#}P \leq 0.01$; $^{***}P \leq 0.001$; and $^{****,\#\#\#\#}P \leq 0.0001$.

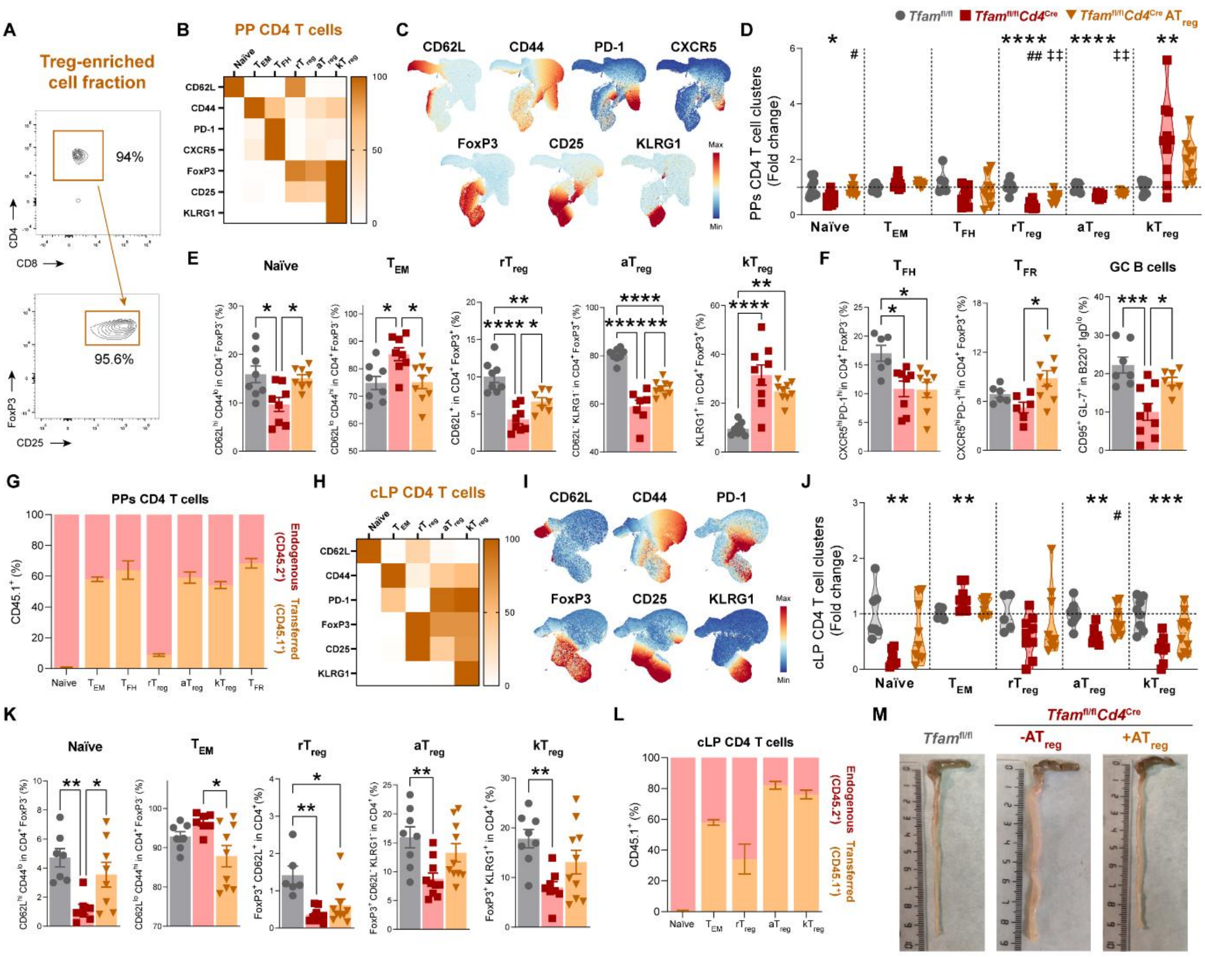

Fig. S10. Adoptive therapy with Treg-enriched cells restores immune homeostasis in the intestine of *Tfam*$^{fl/fl}$*Cd4*^{Cre} mice. (**A**) Percentage of FoxP3^{+}CD25^{+} cells in isolated donor cells after purification. (**B**) Heatmap portraying normalized values of mean fluorescence intensity of each marker in CD4 T cell clusters in Peyer's patches (PPs) of 10-month-old *Tfam*$^{fl/fl}$ and *Tfam*$^{fl/fl}$*Cd4*^{Cre} mice after the adoptive transfer of regulatory T cell (Treg)-enriched cells (ATreg). (**C**) UMAP representation of the expression levels of markers in PP clusters. (**D**) Fold change of PP CD4 T cell clusters compared to control mice (n = 3 to 6). (**E**) Quantification of naïve, effector/memory-like (T_{EM}), resting (r), activated (a), and KLRG1^{+} (k) Tregs in PPs (n = 3 to 6). (**F**) Quantification of T follicular helper (T_{FH}) and regulatory (T_{FR}) cells and germinal center (GC) B cells in PPs (n = 3 to 6). (**G**) Mean chimeric ratio in PP CD4 T cell subsets. (**H**) Heatmap portraying normalized values of mean fluorescence intensity of each marker in in the colonic lamina propria (cLP) clusters. (**I**) UMAP representation of the expression levels of markers in cLP CD4 T cell clusters. (**J**) Fold change of cLP CD4 T cell clusters compared to control mice (n = 3 to 6). (**K**) Quantification of cLP CD4 T cell subsets (n = 3 to 6). (**L**) Mean chimeric ratio in cLP CD4 T cell subsets. (**M**) Representative images of the colon. Data are pooled from N = 2 independent experiments. Data are shown as means ± SEM, where each dot is a biological sample. P values were determined by one-way analysis of variance (ANOVA) with Tukey's multiple comparisons test. *Tfam*$^{fl/fl}$ versus *Tfam*$^{fl/fl}$*Cd4*^{Cre} (*); *Tfam*$^{fl/fl}$*Cd4*^{Cre} versus *Tfam*$^{fl/fl}$*Cd4*^{Cre} ATreg ($^{\#}$); *Tfam*$^{fl/fl}$ versus *Tfam*$^{fl/fl}$*Cd4*^{Cre} ATreg (‡). $^{*,\#}P \leq 0.05$; $^{**,\#\#,\ddagger\ddagger}P \leq 0.01$; $^{***}P \leq 0.001$; and $^{****}P \leq 0.0001$.

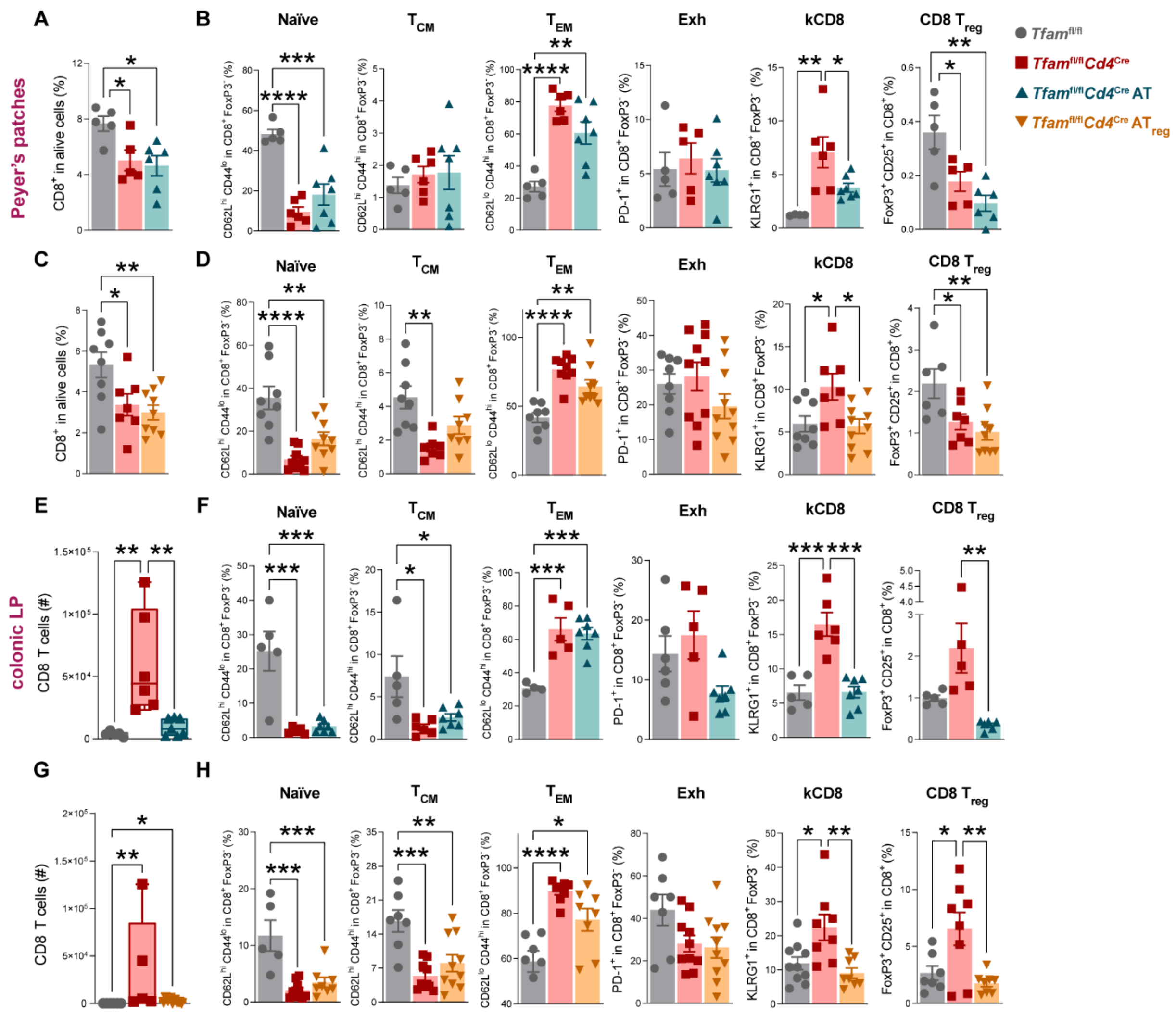

Fig. S11. Analysis of the intestinal CD8 T cell compartment in *Tfam*$^{fl/fl}$*Cd4*^{Cre} mice after adoptive transfer strategies. (**A** and **C**) Percentage of CD8 T cells in Peyer's patches (PPs) of 10-month-old *Tfam*$^{fl/fl}$, *Tfam*$^{fl/fl}$*Cd4*^{Cre} and *Tfam*$^{fl/fl}$*Cd4*^{Cre} mice transferred with (A) competent CD4 T cells (AT) or (C) T regulatory cell (Treg)-enriched cells (ATreg) (n = 3 to 7). (**B** and **D**) Quantification of naïve, central memory (T_{CM}), effector/memory-like (T_{EM}), exhausted (Exh), KLRG1^{+} (kCD8), and regulatory (CD8 Treg) CD8 T cell subsets in PPs of mice after (B) adoptive transfer of competent CD4 T cells (AT) or (D) Treg-enriched cells (n = 3 to 7). (**E** and **G**) Absolute number of CD8 T cells in the colonic lamina propria (cLP) of mice transferred with (E) competent CD4 T cells (AT) or (G) Treg-enriched cells (n = 3 to 7). (**F** and **H**) Quantification of naïve, T_{CM}, T_{CM}, Exh, kCD8, and CD8 Tregs in the cLP of mice after (F) adoptive transfer of competent CD4 T cells (AT) or (H) Treg-enriched cells (n = 3 to 7). Data are (A, B, E, and F) representative of N = 2 or (C, D, G, and H) pooled from N = 2 independent experiments. Data are shown as means ± SEM, where each dot is a biological sample. P values were determined by (A to F, and H) one-way analysis of variance (ANOVA) with Tukey's multiple comparisons test, or (G) Kruskal–Wallis H test with Dunn's multiple comparison test. *$P \leq 0.05$; **$P \leq 0.01$; ***$P \leq 0.001$; and ****$P \leq 0.0001$.

Table S1. Primers for qPCR analysis of the gut microbiota and the intestine of mice.

Gene	Oligonucleotide (FW)	Oligonucleotide (RV)
Enterobacteriaceae	CATTGACGTTACCCGCAGAAGAAGC	CTCTACGAGACTCAAGCTTGC
Lactobacillus sp.	ACCGAGAACACCGCGTTATTT	CATAACTTAACCTAAACAATCAAAGATTGTCT
16S rRNA	TCCTACGGGAGGCAGCAGT	GGACTACCAGGGTATCTAATCCTGTT
Tnf	CTATGTCTCAGCCTCTTCTC	CATTTGGGAACTTCTCATCC
Ifng	CTAGCTCTGAGACAATGAAC	CTCTTTTCTTCCACATCTATGC
S100a8	CAAGGAAATCACCATGCCCTCTA	ACCATCGCAAGGAACTCCTCGA
Serpine1	CCAACATCTTGGATGCTGAA	GCCAGGGTTGCACTAAACAT
Cldn1	TCTACGAGGGACTGTGGATG	TCAGATTCAGCAAGGAGTCG
Ocln	GCTGTGATGTGTGTGAGCTG	GACGGTCTACCTGGAGGAAC
Tpj1	AGGACACCAAAGCATGTGAG	GGCATTCCTGCTGGTTACA
Muc2	CTCCATTGAGTTTGGGAACAT	TTCGGCTCGGTGTTCAGAG
Defa5	CTCCTCTCTGCCCTTGTCCT	GATTTCTGCAGGTCCCAAAA
Defensin	GGTGATCATCAGACCCCAGCATCAGT	AAGAGACTAAAACTGAGGAGCAGC
Crypt1	TCAAGGGCTGCAAAGGAAGAGAAC	TGGTCTCCATGTTCAGCGACAGC
Lyz1	GAGACCGAAGCACCGACTATG	CGGTTTTGACATTGTGTTCGC
Ang4	GGTTGTGATTCCTCCAACTCTG	CTGAAGTTTTCTCCATAAGGGCT
Tff3	TTGCTGGGTCCTCTGGGATAG	TACACTGCTCCGATGTGACAG
Cdkn1a	CTGACAGATTTCTATCACTCC	TTAAGACACACAGAGTGAGG
Tp53	GGGGAGGAGCCAGGCCATCA	CCGCGCCATGGCCATCTACA
Il6	GTCTATACCACTTCACAAGTC	TGCATCATCGTTGTTCATAC
Stat1	GCTTGACAATAAGAGAAAGGAG	CTCGTCATTAATCAGAGTGTTC
Hprt	TCCTCCTCAGACCGCTTTT	CCTGGTTCATCATCGCTAATC
Pp1a	ACGCCACTGTCGCTTTTC	GCAAACAGCTCGAAGGAGAC
B2m	TACATACGCCTGCAGAGTTAAGCA	TGATCACATGTCTCGATCCCAG

Table S2. Optimization of multiple reaction monitor (MRM) condition acquisition modes by direct infusion of 3-nitrophenylhydrazine (3NPH) derivatives.

3NPH-SCFA derivatives	Precursor ion (m/z)	Product ion (m/z)	Fragmentor voltage (V)	Collision energy (eV)
AA (C2)	194.1	**137**<br>46	92	**16**<br>44
PA (C3)	208.1	**137**<br>46	92	**20**<br>48
i-BA/BA (C4)	222.1	**137**<br>107	92	**20**<br>28
2-Me-BA/i-VA/VA (C5)	236.1	**137**<br>107	102	**20**<br>32
2-Me-VA (C6)	250.1	**137**<br>107	102	**20**<br>31

Table S3. Antibodies used for flow cytometry staining.

Marker	Fluorophore	Clone	Reference	Supplier	Dilution
CD45.1	APC Cy7	A20	25-0453-U100	Tonbo Bioscience	200
CD4	APC-Fire 810	GK1.5	100480	Biolegend	400
CD8	PE-Fire 700	53-6.7	100791	Biolegend	400
CD62L	BV711	MEL-14	104445	Biolegend	500
CD44	BV650	IM7	103049	Biolegend	200
CXCR5	PE-Cy7	L138D7	145516	Biolegend	200
PD-1	BV421	29F.1A12	135221	Biolegend	200
CD25	PE-Cy5	PC61	102010	Biolegend	400
KLRG1	BV785	2F1	138429	Biolegend	200
B220	APC-Cy7	RA3-6B2	25-0452-U100	Tonbo Bioscience	200
IgD	V450	11-26c.2a	560869	BD Bioscience	200
CD95	PE-Cy7	Jo2	557653	BD Biosciences	200
GL-7	Alexa Fluor 488	GL7	53-5902-82	eBioscience	200
IgA	PE	mA-6E1	12-4204-82	eBioscience	200
FOXP3	FITC	FJK-16s	11-5773-82	eBioscience	100
CCL5	PE	2E9/CCL5	149104	Biolegend	200
TNF	APC	MP6-XT22	506308	Biolegend	100
IFN-γ	Spark NIR 685	XMG1.2	505862	Biolegend	100

Data file S1. (separate file)
Raw data file.
